# Supplementary material for: Systemic inflammatory markers of visceral leishmaniasis treatment response in East Africa
Source: PLoS Negl Trop Dis. 2026 Feb 27;20(2):e0013749. doi: 10.1371/journal.pntd.0013749 (PMC12965683; doi:10.1371/journal.pntd.0013749)
Supplement: S1 Appendix — (DOCX) [file pntd.0013749.s001.docx]

**Prev PKDL Consortium**

**Ethiopia**

*Department of Medical Parasitology, School of Biomedical and Laboratory Science, College of Medicine and Health Sciences, University of Gondar*

Ayenew Addisu

Asrat M. Hailu

Ayalew Jejaw Zeleke

Nega Dessie

Mulugeta Aemero

*Leishmaniasis Research and Treatment Centre*

Eleni Ayele

Comments from Prof. Asrat for the following lists

Yonael Mulat

Tadele Mulaw

Zemenay Mulugeta

Arega Yeshanew

Roman Melkamu

Armauer Hansen Research Institute, Addis Ababa P.O. Box 1005, Ethiopia.

Menberework Chanyalew

**Kenya**

*Center for Clinical Research, Kenya Medical Research,*

Christine Ichugu

Beatrice Linguli

Daniel Matano

Margaret Mbuchi

Jane Mbui

Jaspher Ndege

Finnley Osuna

Wilson Biwot

Peter Cheboi

Baringo County, Chemolingot Sub County Hospital, African Centre for Community Investment in Health (ACCIH)

Elizabeth Chebet

**UK**

*York Biomedical Research Institute and Hull York Medical School, University of York*

Helen Ashwin

Najmeeyah Brown

Joao Cunha

Paul M. Kaye

Charles Lacey

Rebecca Wiggins

*Bioscience Technology Facility, University of York*

Lesley Gilbert

Karen Hogg

Sally R. James

Peter O’Toole

**Sudan**

*Institute of Endemic Diseases*

Eltahir A.G. Khalil

Ahmed M Musa

Brima M. Younis

*Professor El-Hassan’s Center for Tropical Medicine, Dooka*

Mohammed Alamin

Elmukashfi T. A. Elmukashfi

Amin E.A. Musa

Ala Eldin Mustafa

Ali Noureldein

Mohammed Saeed

Khalid Salman

Ahmed J. Suliman

**Uganda**

*Makerere University*

Alice Bayiyana

Joseph Olobo

*Amudat Hospital*

Patrick Sagaki

Brenda Adiko

Daniel Kalepon

**European Vaccine Initiative**

Flavia D’Alessio

Sophie Houard

Stefan Jungbluth

Odile Leroy

Giovanna Milano

Maria del Mar Castro Noriega

Nicola Viebig

Kimberley Veenstra

**Scientific and Ethical Advisory Committee**

Stephen Cose

Simon Croft

Nirmal K. Ganguly

David Smith
