# Supplementary material for: Systemic inflammatory markers of visceral leishmaniasis treatment response in East Africa
Source: PLoS Negl Trop Dis. 2026 Feb 27;20(2):e0013749. doi: 10.1371/journal.pntd.0013749 (PMC12965683; doi:10.1371/journal.pntd.0013749)
Supplement: S1 Supplementary Information — (PDF) [file pntd.0013749.s002.pdf]

Study Acronym: ImmStat@Cure

# A multicentre observational study to assess immune response status in patients before and after treatment for visceral leishmaniasis

**Protocol version:** 1.8
**Protocol date:** 23rd January 2024
**Sponsor:** University of York
**Clinicaltrials.gov reference**: NCT04342715

Modification History

| Version | Date | Author(s) and details of changes from previous version |
|---|---|---|
| V1.0 | 13.09.19 | None |
| V1.1 | 20.12.19 | Add sites for HEC recruitment and blood draws; minor text changes for clarification (4.4 & 4.5); clarification of exclusion criteria to any previous form of leishmaniasis, and also any of HIV, HBV, HCV; change in blood volume for healthy volunteers (6.1) |
| V1.2 | 23.01.20 | Minor changes including adding Dr Daniel Jeffares as an Investigator, addition of MRC as a funder, language re subjects per site clarified, references to AEs removed, reference to clotting studies removed (but retained as per site specific protocols). |
| V1.3 | 24.01.20 | Clarified healthy control inclusion and exclusion criteria |
| V1.4 | 24.02.20 | Clarification of amount of blood drawn at visits (Table 1 and 4.4, 6.1), addition of Irina Meln as a study coordinator. |
| V1.5 | 24.03.20 | Section 10.3 update regarding relevant University of York Ethics Committee (removal of reference to Hull York Medical School EC). |
| V1.6 | 18.06.20 | Changes to insurance section with removal of no-fault insurance statement. |
| V1.7 | 08.06.23 | Addition of Centre for Tropical Medicine, Doka as a site in Sudan. Section 5.0, section 6.0 - extension of recruitment to children ≤9 years old, site-specific malnutrition scores, increase in blood vol. @D17 visit from 15mL to 20mL for a repeat clinical haematology/biochemistry assessment. Change in EVI coordinators and addition of Dr Karen Hogg as flow cytometry manager. |
| V1.8 | 23-01-24 | Selected sample analyses from all contributing sites to be undertaken at KEMRI (Section 9.7) |

| | |
|---|---|
| Chief Investigator (Ethiopia) | Professor Asrat Hailu Mekuria<br>Dept. of Parasitology,<br>College of Medicine & Health Sciences,<br>University of Gondar,<br>Gondar, Ethiopia<br>Email: hailu_a2004@yahoo.com |
| Chief Investigators (Kenya) | Dr Jane Mbui and Dr Margaret Mbuchi<br>Centre for Clinical Research,<br>Kenya Medical Research Institute,<br>Nairobi, Kenya<br>Email:jmbui@kemri.org<br>Email: Mbuchi.margaret@gmail.com |
| Chief Investigator (Sudan) | Professor Ahmed Mudawi Musa<br>Institute of Endemic Diseases,<br>Department of Clinical Pathology & Immunology,<br>University of Khartoum,<br>Khartoum, Sudan<br>Email: amusa@iend.org |
| Chief Investigator (Uganda) | Professor Joseph Olobo<br>Department of Microbiology<br>School of Biomedical Sciences,<br>College of Health Sciences,<br>Makerere University,<br>Kampala, Uganda<br>Email:oloboj@yahoo.co.uk |
| Chief Investigator (York) | Professor Paul Kaye,<br>York Biomedical Research Institute<br>Hull York Medical School,<br>University of York,<br>York, UK<br>Email: paul.kaye@york.ac.uk |
| Study Coordinators | Dr Flavia Dalessio and Dr Kimberly Veenstra,<br>European Vaccine Initiative,<br>Universitätsklinikum Heidelberg,<br>Heidelberg, Germany<br>Email: flavia.dalessio@euvaccine.eu<br>Email: kimberly.veenstra@euvaccine.eu |
| Sponsor clinical representative | Professor Charles Lacey<br>York Biomedical Research Institute<br>Hull York Medical School<br>University of York, UK<br>Email: charles.lacey@hyms.ac.uk |

| | |
|---|---|
| Statistical Investigators | TBC |
| Flow Cytometry Manager | Dr Karen Hogg<br>Department of Biology<br>University of York, UK<br>Email karen.hogg@york.ac.uk |
| Site Investigators – Ethiopia | Dr.Rezika Mohammed and Dr.Helina Fikire |
| Site Investigator - Uganda | Dr Patrick Sagaki |
| Site Investigator - York | Dr Daniel Jeffares |

| | |
|---|---|
| Study Manager | Dr Rebecca Wiggins<br>York Biomedical Research Institute<br>Hull York Medical School<br>University of York, UK<br>Email: rebecca.wiggins@york.ac.uk |
| Laboratories | Department of Parasitology and the Leishmaniasis Research & Treatment Centre, College of Medicine & Health Sciences, University of Gondar, Ethiopia<br><br>Kenya Medical Research Institute<br>P.O. Box 20778 KNH 00202, Nairobi, Kenya<br><br>Institute of Endemic Diseases,<br>Department of Clinical Pathology & Immunology,<br>University of Khartoum, Sudan<br><br>Department of Microbiology,<br>School of Biomedical Sciences,<br>College of Health Sciences,<br>Makerere University,<br>Kampala, Uganda<br><br>York Biomedical Research Institute<br>University of York<br>York YO10 5DD, UK |
| Study Sites | Leishmaniasis Research & Treatment Centre,<br>University of Gondar, Gondar, Ethiopia<br><br>Kimalel and Chomolingot Health Centres,<br>Baringo County, Kenya<br><br>Institute of Endemic Diseases, |

| | |
|---|---|
| | Department of Clinical Pathology & Immunology, University of Khartoum, Sudan<br><br>Al Hassan Centre for Tropical Medicine Doka, Gedarif State, Sudan<br><br>Amudat Hospital, Amudat, Uganda |
| Sponsoring Institution | University of York, York, UK |
| Funding | European & Developing Countries Clinical Trials Partnership (EDCTP), through the project award PREV_PKDL (Ref No. RIA2016V-1640)<br>Medical Research Council |

**Description of the Clinical Sites and Laboratories**

**In Ethiopia:**
Patient recruitment and study visits will take place in the University of Gondar (UoG). Teaching will take place in Hospital, Gondar, Ethiopia. All haematological and biochemical screening and safety tests will be conducted in the University of Gondar. Samples for immunological assays will be processed and stored initially in the University of Gondar. Some samples will be subsequently shipped under MTA to York Biomedical Research Institute, University of York, UK and KEMRI, Kenya. Healthy endemic controls will be recruited from the Gondar region and blood drawn at University of Gondar.

**In Kenya:**
Patient recruitment, study visits, haematological and biochemical screening and safety tests will take place at Kimalel and Chemolingot Health Centres, Baringo County, Kenya. Samples for immunological assays will be processed and stored initially at the Kimalel and Chemolingot Health Centres and then transported to and processed in Kenya Medical Research Institute (KEMRI). Some samples will be subsequently shipped under MTA to the York Biomedical Research Institute, University of York, UK. Healthy endemic controls will be recruited from the Nairobi region and blood drawn at KEMRI. Some samples from study sites in Uganda, Sudan and Ethiopia will be shipped to and analysed in the laboratories at KEMRI. These samples will be transported under an MTA between KEMRI and each study site.

**In Sudan:**
Patient recruitment, study visits, haematological and biochemical screening and safety tests will take place at Soba Hospital, Khartoum, the Institute for Endemic Diseases (IEND), the El Hassan Centre for Tropical Medicine, Doka, Gedarif State and the University of Khartoum, Sudan. Samples for immunological assays will be processed and stored initially in the IEND, Khartoum or the Centre for Tropical Medicine, Doka. Some samples will be subsequently shipped under MTA to the York Biomedical Research Institute, University of York, UK, and KEMRI, Kenya. Healthy

endemic controls will be recruited from any of the above regions and blood drawn at Soba Hospital, IEND or the Centre for Tropical Medicine.

**In Uganda:**
Patient recruitment, study visits, haematological and biochemical screening and safety tests will take place at Amudat Hospital, Amudat, Uganda. Samples for immunological assays will be processed and stored initially in Amudat Hospital then transported to the Department of Microbiology, Makerere University (MU). Some samples will be subsequently shipped under MTA to the York Biomedical Research Institute, University of York, UK and KEMRI, Kenya. Healthy endemic controls will be recruited from the Kampala region and blood drawn at MU.

<u>Definitions used in this protocol:</u>

Chief Investigator (CI): Takes ultimate responsibility for the design, conduct, analysis and reporting of the clinical study.

Study Coordinators: Intermediary between funder and PREV_PKDL project partners, in charge of overall supervision of the PREV_PKDL project.

Sponsor Clinical representative: A member of the clinical team with designated responsibilities, who advises the sponsor on the conduct of the study.

Statistical Investigator: An individual member of the statistical team with designated responsibilities, who in terms of the clinical study is under the overall supervision of the Chief Investigator (York).

Site Investigator: An individual member of the study team working at one of the study sites and implementing the protocol under the direction of the Site Chief Investigator.

**Protocol Signature Page**

The signature below confirms agreement by the individual authorised by the Sponsor and responsible for signing the clinical study agreement, that the study ImmStat@Cure will be conducted in accordance with this protocol and GCP and ICH guidelines. Any amendments to this protocol that have a direct influence on the volunteers in the study will be approved by the relevant ethics committees before implementation.

We, the Chief Investigators, agree to allow sponsor monitor and auditors, full access to all medical records at the research facility for volunteers screened or enrolled in the study.

We agree to maintain all study documentation until the Sponsor consents to disposal of files in writing.

We have read and understood the information in the study protocol and will ensure that all colleagues and employees assisting in the conduct of the study are informed about the obligations incurred by their involvement in the study.

We will ensure all studies are conducted in accordance with the appropriate Site Specific Protocols.

**Signatures:**

| Name of Chief Investigator (s) | Institution | Sign./initials | Date |
|---|---|---|---|
| Professor Asrat Hailu | | | |
| Dr. Jane Mbui | | | |
| Dr. Margaret Mbuchi | | | |
| Professor Ahmed Mudawi Musa | | | |
| Professor Joseph Olobo | | | |
| Professor Paul Kaye | | | |
| Professor Charles Lacey | | | |

**Confidentiality Statement**

This document contains confidential information that must not be disclosed to anyone other than the Sponsor, the Investigator Team, and members of the Research Ethics Committee. This information cannot be used for any purpose other than the evaluation or conduct of the clinical investigation without the prior written consent of the Chief Investigators - Professor Asrat Hailu Mekuria, Dr Jane Mbui, Dr Margaret Mbuchi, Professor Ahmed Mudawi Musa, Professor Joseph Olobo, Professor Paul Kaye, and Professor Charles Lacey.

**Serious breach**
**Within 24h of becoming aware of a serious breach, notify the Sponsor**
**+44 7802 242996 email: charles.lacey@hyms.ac.uk / rebecca.wiggins@york.ac.uk**

Page Number

Contents


**1.0 STUDY SUMMARY ..... 13**

*2.1 Background and clinical unmet need ..... 14*

*3.1 Primary Objective ..... 17*

*3.2 Secondary Objectives ..... 17*

**4.0 STUDY DESIGN ..... 18**

*4.1 Study Centres ..... 18*

*4.2 Study Design ..... 18*

*4.3 Study Volunteers ..... 18*

*4.4 Study procedures ..... 18*

*4.5 Duration of Study ..... 18*

**5.0 RECRUITMENT AND WITHDRAWAL OF STUDY VOLUNTEERS ..... 19**

*5.1 Number and Source of Patient Volunteers ..... 19*

*5.2 Inclusion and Exclusion Criteria ..... 19*

Patient Inclusion Criteria ..... 19

Patient Exclusion Criteria ..... 19

Healthy endemic control inclusion criteria ..... 20

Healthy endemic control exclusion criteria. ..... 20

*5.3 Screening Procedures and Investigations. ..... 21*

*5.4 Eligibility ..... 22*

*5.5 Procedures in relation to adverse events during VL treatment ..... 22*

**6.0 TREATMENT OF STUDY VOLUNTEERS ..... 22**

*6.1 Study procedures ..... 22*

*6.2 Expected risk and discomfort ..... 23*

*6.3 Additional Visits ..... 26*

**7.0 WITHDRAWAL, PREGNANCY, MONITORING, AND STUDY TERMINATION ..... 26**

*7.1 Withdrawal of Volunteers ..... 26*

*7.2 Pregnancy ..... 26*

*7.3 Monitoring ..... 26*

*7.4 Sponsor ..... 26*

**8.0 STATISTICS ..... 26**

*8.1 Sample Size ..... 26*

*8.2 Statistical Analysis Plan (SAP) ..... 27*

**9.0 MANAGEMENT OF DATA, SAMPLES AND TRIAL PROCEDURES........................27**
*9.1 Source Data and Case Report Forms (CRFs)........................27*
*9.2a Screening Log........................27*
*9.2b Volunteer Log........................28*
*9.3 Access to Data........................28*
*9.4 Data Protection........................28*
*9.5 Archiving of Data........................28*
*9.6 Confidentiality........................28*
*9.7 Management of Biological Samples........................28*
*9.8 End of the Study........................29*
*9.11 Trial Management........................30*
**10.0 ETHICS and ethical considerations........................30**
*10.1 Declaration of Helsinki........................30*
*10.2 ICH Guidelines for Good Clinical Practice........................30*
*10.3 Research Ethics Committee (REC)........................30*
*10.4 Volunteer Confidentiality........................31*
*10.5 Regulatory and ethical approvals........................31*
*10.6 Informed Consent Form........................31*
*10.7 Risks........................32*
*10.8 Benefits........................32*
*10.9 HIV Testing........................32*
*10.10 Reimbursement........................32*
**11.0 REGULATORY AND GOVERNANCE ISSUES........................33**
*11.1 Required approvals........................33*
*11.2 Amendments........................33*
*11.2 GCP, and GLP Compliance........................33*
**12.0 INDEMNITY........................33**
***12.1 Negligent Harm**........................33*
**13.0 FINANCE........................34**
*13.1 Financing........................34*
*13.2 Reimbursement for Volunteers........................34*
**14.0 PUBLICATION........................34**
**16.0 REFERENCES........................35**
**Appendix 1: Source Data Definition........................38**

**ABBREVIATIONS**

| | |
|---|---|
| ALT | Alanine Aminotransferase |
| AST | Aspartate Aminotransferase |
| BMI | Body Mass Index |
| BNF | British National Formulary |
| CI | Chief Investigator |
| CL | Cutaneous Leishmaniasis |
| CRF | Case Report Form |
| CSG | Clinical Study Group |
| EAR | East African Region |
| FBC | Full Blood Count |
| GCP | Good Clinical Practice |
| GLP | Good Laboratory Practice |
| HCV | Hepatitis C virus |
| HEC | Healthy endemic controls |
| HIV | Human Immunodeficiency Virus |
| HYMS | Hull York Medical School |
| ICH | International Conference on Harmonisation |
| IEND | Institute of Endemic Diseases, Khartoum |
| LFT | Liver Function Test |
| LLN | Lower Limit of Normal |
| MTA | Material Transfer Agreement |
| ml | Millilitre |
| OTC | Over the Counter |
| PI | Principal Investigator |
| PKDL | Post kala-azar dermal leishmaniasis |
| PM | Paromomycin |
| R&D | Research and Development |
| RDT | Rapid Diagnostic Test |
| REC | Research Ethics Committee |
| RSG | Research Steering Group |
| SAP | Statistical Analysis Plan |
| SD | Standard Deviation |
| SMF | Study Master File |

| | |
|---|---|
| SOP | Standard Operating Procedure |
| SSF | Site Study File |
| SSG | Sodium stibogluconate |
| | |
| Th | T helper |
| TL | Tegumentary Leishmaniasis |
| | |
| U&E | Urea and Electrolytes |
| ULN | Upper Limit of Normal |
| VL | Visceral Leishmaniasis |
| WHO | World Health Organisation |
| µg | microgram |

## 1.0 STUDY SUMMARY

| | |
|---|---|
| **Title** | A multicentre observational study to ascertain immune status of patients before and after treatment for visceral leishmaniasis. |
| **Study Centres** | University of Gondar,<br>Gondar, Ethiopia<br><br>Kimalel and Chemolingot Health Centres,<br>Baringo County, Kenya<br><br>Institute for Endemic Diseases andCentre for Tropical Medicine<br>University of Khartoum,<br>Khartoum, and<br>Al Hassan Centre for Tropical Medicine,<br>DokaSudan<br><br>Amudat Hospital,<br>Amudat, Uganda |
| **Study Identifier** | ImmStat@Cure |
| **Design** | Observational |
| **Population** | 9-50 years old; M or F; VL patients undergoing treatment with SSG/PM<br>9-50 years old; M or F; healthy controls |
| **Sample Size** | 40 patients and 30 controls per site |
| **Follow-up duration** | 6 months from end of treatment |
| **Planned Trial Period** | 36 months |
| **Primary Objective** | To assess systemic and skin immune responses and parasite load in patients before, and at end of treatment for visceral leishmaniasis |
| **Secondary Objectives** | 1. To identify systemic and local immune correlates of treatment outcome.<br>2. To evaluate parasite genotype in relation to immune response characteristics<br>3. To compare immunology and parasitological data across each site and determine possible correlates of progression to PKDL |

## 2.0 BACKGROUND AND RATIONALE

### 2.1 Background and clinical unmet need

*The Leishmaniases*

The leishmaniases are poverty-related neglected diseases with a major impact on health worldwide [1]. They affect the poorest of the poor and present a severe barrier to socio-economic development. Caused by infection with one of several species of *Leishmania* parasite, these diseases occur in 98 countries worldwide and can be broadly classified as tegumentary leishmaniases (TL; affecting the skin and mucosa) and visceral leishmaniasis (VL; affecting internal organs). Worldwide, one billion people are thought to be at risk, with 600,000-1,000,000 reported cases of TL and 50,000-90,000 reported cases of VL occurring each year. Whereas TL is chronic and non-life-threatening, VL is responsible for 20,000-30,000 deaths per year, second only to malaria amongst parasites with regard to mortality. Collectively, approximately 2.4M disability-adjusted life years are lost to the leishmaniases. **No vaccines are currently licensed for any form of human leishmaniasis and the drug arsenal is limited and increasingly compromised by drug resistance.**

VL (or kala azar) is a fatal systemic disease most commonly resulting from infection with *L. donovani* or *L. infantum*, but occasionally due to other species, e.g. *L. tropica*. Ninety percent of the VL burden lies in five countries (India, Bangladesh, Nepal, Sudan and Brazil). With the reduction in cases in South Asia, associated with a tri-national elimination campaign (single dose AmBisome treatment and use of indoor residual spraying), **East Africa Region (EAR) may now harbour the greatest burden of VL of any region, reporting an annual number of VL cases of 8,000-13,000, and an average death rate of 4-11%.** Eighty percent of VL cases in Sudan are reported from Gedaref State, but new foci are appearing, e.g. around the White Nile. Ethiopia also carries a significant disease burden, with VL widely spread geographically. In Kenya, VL has been reported in six counties namely West Pokot County (bordering Uganda), Baringo County, Marsabit County, Wajir County and Turkana County (bordering South Sudan). The highest burden is usually reported from West Pokot County with an annual average incidence of 400 cases/year. In Uganda, VL is found in eastern Uganda bordering Kenya in the West Pokot region, particularly in Amudat District (Karamoja Region).

In contrast to South Asia, AmBisome is less effective in the EAR, where first-line treatment is currently sodium stibogluconate combined with paromomycin (SSG/PM) given for 17 days. Approximately 10% of VL patients fail first-line therapy [2]. New combination therapies including oral miltefosine are being explored under the auspices of the Drugs for Neglected Diseases initiative in partnership with countries comprising the Leishmaniasis East Africa Platform (Ethiopia, Kenya, Sudan and Uganda). HIV co-infection, associated with a poor prognosis, is of significance in Ethiopia but less so elsewhere in the EAR, and this brings additional challenges for patient management [3, 4].**The effectiveness of these drug regimens in reducing infectiousness to sand flies and preventing the**

**development of post kala azar dermal leishmaniasis (PKDL) remains unclear.**

PKDL is a severe and chronic form of tegumentary leishmaniasis that usually develops after treatment for VL caused by *L. donovani*, but which can occur in the absence of previous VL or concomitant with VL therapy (more specifically called *para*-kala azar dermal leishmaniasis [5-8].) PKDL is found in South Asia and predominantly in Sudan within EAR, where 3000-5000 cases per year are estimated to occur. PKDL always affects the face and often the arms and trunk [9, 10]. Hence, PKDL significantly affects quality of life, is often mistaken for leprosy, and can result in stigmatisation that has lifetime impact. Many PKDL patients do not undergo treatment (see below) and these individuals likely contribute to the maintenance of VL in communities, as parasites persist for many months or even years in skin lesions and can be acquired by the sand fly vector [11]. As with VL, PKDL/HIV coinfection can occur, with more severe PKDL or recrudescence common even with anti-leishmanial and anti-retroviral therapy [7]. **Our understanding of the pathogenesis of PKDL is poor and the lack of tools to prevent and/or treat PKDL is now a well-recognised challenge to VL elimination** [12]**.**

Strikingly for both VL and PKDL, the natural history of disease varies geographically, with scientific and practical implications. For example, in Gedaref (Sudan) and Gondar (Ethiopia), PKDL is more common, treatment response to paromomycin is poor, single dose AmBisome is ineffective and there is evidence of parasite diversity (by multilocus typing or RAPD PCR [13]). However, in southern Ethiopia (Arba Minch), single dose AmBisome was effective [14]. In contrast, in Kimalel (Kenya) and Amudat (Uganda), PKDL is rare or absent, multiple dose AmBisome was effective [15] and there is limited parasite diversity. These differences translate into treatment practice. For example, in South Asia, when PKDL is persistent, all cases are treated with miltefosine or AmBisome. In Sudan, AmBisome is reserved for persistent or severe cases of PKDL due to the risks of nephrotoxicity and hypokalemia [16].

Differences in immunopathology are thought to play a role in the diversity of PKDL natural history, although to date only relatively low-resolution analyses (i.e. measurement of a few cytokines or cell types) have been performed on clinical material. Parasite genotypic diversity in South Asia has been examined at high molecular resolution by next generation sequencing [17], but beyond the links to drug resistance, its role in host phenotypic heterogeneity is yet to be established. **It is not known why PKDL develops at different rates and with different frequency in patients treated for VL in different countries across EAR.**

*Immune responses in VL pre and post treatment*

The immune response during VL has been studied extensively in rodent models and in humans [18, 19]. The main effector mechanism against intracellular *Leishmania* amastigotes is phagocyte activation leading to the generation of toxic oxygen species as well as metabolic stress. These effector mechanisms are regulated by cytokines produced both by infected phagocytes themselves and also by *Leishmania*-specific T lymphocytes. Both $CD4^+$ T cell and $CD8^+$ T cells have been shown to contribute to host resistance,

primarily through the production of interferon-$\gamma$. Other cytokines as well as cytotoxicity may also play a host protective role. Poor effectiveness of cell mediated immunity is thought to be due to a combination of regulatory or suppressive influences, including those mediated by alternate T cell and phagocyte-derived cytokines (e.g. IL-10 [20]), by antigen-specific regulatory T cells or through T cell exhaustion [21, 22]. The latter arises when T cells engage specific ligands on infected phagocytes (e.g. checkpoint ligands such as PD-L1) Understanding how this process occurs in the context of the tissue microenvironment has been instrumental in developing new treatments for cancer [23], but poorly explored in the context of infectious disease. The advent of transcriptomics has allowed greater insights into the mechanisms of immunity in VL in India [24], Brazil [25] and Sudan [26], but has yet to be applied across the disease spectrum in EAR or in relation to PKDL. In Indian PKDL, T cell exhaustion has also been demonstrated [27], along with various abnormalities in skin immune status, including the development of M2-phenotype macrophages and monocytes [28]. UV exposure has been cited as a possible immune modulator involved in the pathogenesis of PKDL [6, 29] and IL-10 in healthy skin of VL patients was associated with PKDL development in Sudan [30], but this study has not been extended. **The immune mechanisms that pre-dispose to PKDL are unclear and may be best addressed by comparative studies of VL patients in regions where PKDL does and does not exist.**

*Return to homeostasis after treatment*

It is recognised that after successful treatment for VL, there is a degree of normalisation of host immune status and a restoration of antigen-specific recall responsiveness. However, the extent to which the broader impacts of disease revert to normal are unknown. Poor restoration of homeostasis has been previously recognised as an important predictor of relapse. For example, the extent of residual splenomegaly at the end of treatment was found to be a significant risk factor for relapse in EAR [31]. In rodent models of drug treatment, whilst there is restoration of some immune parameters after parasitological cure, (as judged by transcriptomic analysis), many indicators remain of systemic abnormalities in immune, metabolic and tissue remodelling processes (Ashwin et al, manuscript submitted), but this has not been systematically studied in humans. **It is important to understand the extent to which immune homeostasis is restored after therapy, in order to understand the determinants of long-term treatment failure, whether treated patients remain immunocompromised and at risk of other secondary infections, and whether this impacts on progression to PKDL.**

*Silent skin infection during VL*

Although the clinical manifestations of VL are systemic, there is now ample evidence that parasites may also adopt a skin residency. In both canine VL and in rodent models of VL, skin parasites are detectable by both molecular and histological method and appear to play an important role in parasite transmission, particularly under circumstances where blood parasitemia is limiting [32, 33]. In human VL, parasite antigen was detected in the skin of patients with VL in Sudan [30]. Xenodiagnosis studies ongoing in India and Bangladesh indicate that skin parasite load may also be indicative of host infectiousness to the sand fly vector [11]. Through the use of a new minimally invasive microbiopsy technique, parasite DNA has been identified in the skin of both symptomatic VL patients and a proportion of

endemic controls [34]. In this study, 101/181 seropositive asymptomatic people living in an endemic region of southern Ethiopia, of which only 22 had previously been treated for VL, were parasite positive by microbiopsy, indicating a widespread occurrence of skin parasites in the absence of clinical disease.

The identification of a population of parasites in the skin poses a number of new questions important for patient management, disease control and for understanding the aetiology of PKDL. These include: i) does skin parasite load and the associated local immune response change after SSG/PM treatment; ii) are parasite genotypes the same in viscera and skin or do skin parasites reflect genetic adaptation for transmission; iii) is skin parasite load and / or local immune response predictive of development of PKDL and can this explain geographical differences and provide correlates of PKDL development

**There have been no studies addressing the immunology related to skin parasitism during VL, or how this response may change after treatment, despite increasing evidence that parasites may reside in skin throughout the disease course and be responsible for transmission.**

## 3.0 OBJECTIVES

### 3.1 Primary Objective

To assess systemic and skin immune responses and parasite load in patients before, and at end of treatment for visceral leishmaniasis.

Outcomes: Data on immune cell phenotypes and function in peripheral blood and tissue, as determined using standard methods in immunology (e.g. flow cytometry, transcriptomics and histopathology) and parasite quantitation (e.g. PCR, histopathology); qualitative and quantitative analysis of data across patient cohorts and between patients and controls.

### 3.2 Secondary Objectives

1. To identify systemic and local immune correlates of treatment outcome
2. To evaluate parasite genotype in relation to immune response characteristics
3. To compare immunological and parasitological data across each site and determine possible correlates of progression to PKDL

Outcomes: Data on immune cell phenotypes and function in peripheral blood and tissue as determined using standard methods in immunology (e.g. flow cytometry, transcriptomics and histopathology) and parasite quantitation (e.g. PCR, histopathology).Qualitative and quantitative analysis of data across patient cohorts and between patients and controls.

## 4.0 STUDY DESIGN

### 4.1 Study Centres

This multicentre clinical study will be carried out at the Institute for Endemic Diseases, University of Khartoum, Khartoum, Sudan, the University of Gondar, Gondar, Ethiopia, Amudat Hospital, Amudat, Uganda and the Kimalel and Chemolingot Health Centres, Baringo County, Kenya.

### 4.2 Study Design

This is an observational study of the immune response and parasite load in VL patients before and after treatment with SSG/PM.

### 4.3 Study Volunteers

Forty patients diagnosed with VL from each site will be included in the study. Patients will be willing and able to adhere to the study procedures and to give written informed consent. For more details refer to section 5.0. All patients will have been clinically and parasitologically confirmed as having VL and will receive one round of SSG/PM treatment as per normal clinical practice and regional guidelines. Thirty healthy endemic controls will also be studied from each country.

### 4.4 Study procedures

For patients, confirmation of VL will be made by visual identification of parasites in stained tissue smears according to recommended diagnostic practice (using bone marrow, spleen or lymph node aspirates as clinically indicated and as per local procedures). Parasites will be isolated from residual tissue aspirate and genotyped. Where tissue aspirate is not a recommended or currently performed diagnostic procedure, parasites will be isolated where possible from blood and diagnosis confirmed by PCR.

The study requires two additional blood draws (to a maximum of 15ml), one taken before treatment and the second taken at the end of treatment.

The study also requires two 3mm skin biopsies (taken from the back of the neck/shoulder), one taken prior to the beginning of treatment and the second taken at the end of treatment. Biopsies will be taken under local anaesthesia. Patients refusing skin biopsies can still be recruited into the study.

For healthy controls, 23ml of blood will be taken on a single occasion.

### 4.5 Duration of Study

Potential participants will be consented will be consented to take part in the study with donation of blood samples and skin biopsies before treatment of VL. A follow up visit at 6 months will assess development of PKDL.

Healthy *Leishmania* seronegative controls will be recruited from the local area. No formal follow up will be required for these volunteers.

## 5.0 RECRUITMENT AND WITHDRAWAL OF STUDY VOLUNTEERS

### 5.1 Number and Source of Patient Volunteers

We aim to recruit 40 patients from each site who will be treated for VL using a standard regimen of SSG/PM. Volunteers will be recruited by active case detection and through self-attendance at study hospitals.

30 healthy endemic controls will also be recruited e.g. from urban city areas in each country.

### 5.2 Inclusion and Exclusion Criteria

This study will be conducted on samples obtained from VL patients that meet the following inclusion and exclusion criteria.

**Patient Inclusion Criteria**

The patients must be:

- Aged 9 to 50 years on the day of diagnosis
- Have confirmed diagnosis of VL and be judged suitable for treatment using a standard regimen of SSG/PM
- Willing and able to give written informed consent
- For children aged 9 to 11 years, and adolescents aged 12 to 17 years on the day of screening, written informed consent from a parent must be obtained in addition to assent from the patient
- Without any other significant health problems as determined by medical history, physical examination, results of screening tests and the clinical judgment of a medically qualified Clinical Investigator
- Negative for malaria on blood smear
- Judged, in the opinion of a medically qualified Clinical Investigator, to be able and likely to comply with all study requirements as set out in the protocol

**Patient Exclusion Criteria**

The patient may not enter the study if any of the following apply:

- Has HIV, HBV or HBC
- Has previously had any form of leishmaniasis
- Pregnancy or lactating mothers
- Any confirmed or suspected immunosuppressive or immunodeficient state, including; asplenia; recurrent, severe infections and chronic (more than 14 days) immunosuppressant medication within the past 6 months

- Tuberculosis, leprosy, or severe malnutrition in adults, adolescents and children according to site-specific definitions as follows:

Table 1: Adult malnutrition indices per site

| Method | KEMRI | Gondar | Uganda | Sudan |
|---|---|---|---|---|
| **BMI c/o** | <16.0 | N/A | <18.5 | ≤18.5 |
| **MUAC c/o** | N/A | < 170mm | N/A | N/A |

Table 2: Child and adolescent malnutrition indices per site

<table>
<tr><th>Method</th><th>KEMRI</th><th colspan="2">Gondar</th><th>Uganda</th><th>Sudan</th></tr>
<tr><td>BMI c/o</td><td>Z score cut-off <2 SD</td><td colspan="2">N/A</td><td>Z score cut-off <2 SD</td><td>Z score cut-off <2 SD</td></tr>
<tr><td>MUAC c/o</td><td>N/A</td><td>Height (cm)<br>130-<140<br>140-<150<br>150-<160<br>160-<170<br>>170</td><td>Cut-off (mm)<br><135<br><145<br><150<br><155<br><160</td><td>N/A</td><td>N/A</td></tr>
</table>

- Any other significant disease, disorder or finding, which, in the opinion of a medically qualified Clinical Investigator, may influence the result of the study, or the volunteer's ability to participate in the study
- Unlikely to comply with the study protocol

**Healthy endemic control inclusion criteria**

The volunteer must be:

- Aged 9 - 50 years
- Willing and able to give written informed consent
- Seronegative for *Leishmania* antigens using rk39
- For children aged 9 – 11 years, and adolescents aged 12 to 17 years, written informed consent from a parent must be obtained in addition to assent from the patient
- Negative for malaria on a blood smear
- Willing to undergo screening for HIV, Hepatitis B, and Hepatitis C
- For females only, willing to undergo a urine pregnancy test on the day of screening

**Healthy endemic control exclusion criteria.**

The volunteer may not enter the study if any of the following apply:

- Fever, recent vaccination, significant general medical illness incl. TB, malaria, HIV
- Has past history of any form of leishmaniasis
- Has HIV, HBV, HCV, or if female pregnant

**5.3 Screening Procedures and Investigations.**

The general eligibility criteria will be assessed prior to conducting informed consent:

Prior to VL diagnosis, the volunteer will be fully informed of the purpose of the study, the potential risks and obligations and the additional procedures that will be performed if they are diagnosed with VL. They will be given written material and allowed to consider their recruitment into the study. The Chief Investigator (or a study physician in accordance with the delegation log) has both an ethical and a legal responsibility to ensure that each volunteer being considered for inclusion in the study is given a full explanation of the study.

To ensure informed consent, volunteers will be told by a member of the study team that the additional blood sample and skin biopsies are being taken for research purposes to potentially help others with this disease in the future and that their care and treatment will not be affected by whether they agree to participate or not.

Informed consent will include an explanation of:

- why parasites are being isolated from their diagnostic biopsy
- why blood samples and biopsies are being taken
- what the procedures entail
- the potential risks
- the duration of the procedures
- the expected cosmetic outcome of the biopsy procedure
- what these tests mean for the patient’s treatment

If the volunteer is still willing and wishes to participate, they will be asked to sign and date three copies of the consent form - one for the volunteer to keep, one to be stored in the volunteer’s case record form (CRF) and one to be placed in the Study Site File.

There will be separate consent options to allow recruitment of patients who are willing to give blood samples but not willing to provide skin biopsies.

Consent will also include the option to allow residual material to be re-used for other research purposes.

After informed consent has been obtained, additional investigations above standard care will include:

- Collection of 15mL blood sample pre-treatment and at end of treatment for research use

- Collection of 5mL blood sample at end of treatment for clinical haematology/biochemistry
- Collection of 3mm punch biopsy (if subject consents) pre-treatment and at end of treatment
- Use of residual diagnostic spleen / bone marrow / LN biopsy (where taken) for culture of parasites/isolation of parasite DNA

Each volunteer who enters the study by signing a copy of the consent form will be assigned an Identification Number. These numbers will not be reassigned. A log of screened subjects with their identification number and demographics will be maintained and kept in their clinical notes to track volunteers.

**5.4 Eligibility**

A medically qualified Clinical Investigator must confirm eligibility for each volunteer based on the screening procedures, including findings from clinical histories, examinations, laboratory results. This must be documented in the CRF. If the volunteer is deemed to be eligible, the additional samples can be taken.

If for any reason the volunteer is considered a screen failure the volunteer will be informed by a Clinical Investigator, notified of all of their results and the reason for the screen failure. Such patients will be directed to the VL treatment facility.

**5.5 Procedures in relation to adverse events during VL treatment**

This is an observational study in that it utilises standard practice for clinical investigations and treatment within each centre of the four participating countries. The objectives of the study will therefore be fulfilled by the immunological and parasitological data arising from the various clinical specimens being studied. Therefore, we will not measure adverse events in the same manner as if this were an Investigational Medicinal Product trial. However, each site will produce a site-specific SOP detailing their standard diagnostic procedures and standard treatment for VL. Sponsor Representatives will visit each site during, and at the end of the study, and conduct a Clinical Audit of the care of study participants, detailing adherence to the site-specific VL Diagnosis and Treatment SOP, and any serious morbidity or mortality arising during the course of the study. Serious adverse events arising as a consequence of drug treatment of VL should be reported to the national Medicines Agencies as per normal clinical practice.

**6.0 TREATMENT OF STUDY VOLUNTEERS**

**6.1 Study procedures**

**Patients**

Procedures will be prior to commencement of VL treatment and after the end of treatment. Additional procedures or laboratory tests may be performed, at the discretion of a Clinical Investigator if clinically required.

Prior to treatment and after the end of treatment, additional samples to routine care will include: i) a 15mL venous blood sample for transcriptomic profiling (2.5mL), plasma / PBMC analysis (10mL), whole blood antigen recall assays (2.5mL), blood for clinical haematology and biochemistry at the end of treatment (5mL), and ii) a 3mm full thickness skin biopsy (for transcriptomics and protein profiling and parasite identification). In addition, residual spleen / BM or LN aspirate performed for diagnostic purposes will be used for parasite culture and DNA extraction and / or tissue RNA-Seq.

The windows of compliance with the protocol for these procedures are indicated in the schedule, Table 1.0.

**Healthy volunteers**
Healthy volunteers will be required to provide 15mL of blood for the investigations in this study, and 3mL of blood for blood-borne viruses and malaria screening. They will also be asked to provide an additional 5mL of blood in order to conduct routine haematology and biochemistry tests, which will benefit them in terms of being a “free” health check as part of compensation for being in the study. The total blood volume for healthy volunteers at their visit will be 23mL.

## 6.2 Expected risk and discomfort

*Blood samples:*
Patients: Patients will be undergoing venepuncture in order to provide blood for routine biochemistry and immunological tests used for the diagnosis of VL and end of treatment evaluation. Risk and discomfort associated with venepuncture may include local bruising, pain and occasional light headedness or fainting. The additional 15mL of blood required for this study will be taken after diagnosis is confirmed. There are negligible additional risks, discomfort or AEs associated with this procedure.

Healthy volunteers: Risk and discomfort associated with venepuncture may include local bruising, pain and occasional light headedness or fainting.

Blood samples will be processed for analysis according to the appropriate laboratory SOP.

*Skin biopsy:*
Patients: A 3mm full thickness punch biopsy will be taken after application of local anaesthesia. Normally, pressure applied to the biopsy site will be sufficient to limit any bleeding, but in some cases a suture may be required. If residual local pain persists, an appropriate analgesic will be provided. The biopsy may leave a small scar at the site. Therefore, biopsies will be taken from a site with minimal cosmetic impact should scaring occur e.g. the back of the neck / shoulder. The second biopsy will be performed similarly at a

site close to but not overlapping with the first biopsy site. No cumulative effects from the two biopsies are expected.

Healthy volunteers: Not applicable.

Biopsies will be processed for analysis according to the appropriate laboratory SOP.

*Diagnostic aspirate:*
Patients: Only residual material from a standard diagnostic biopsy will be used. Hence there is no additional risk or discomfort for the patient. Aspirates will be processed for analysis according to the appropriate laboratory SOP.

Healthy volunteers: Not applicable.

**Outpatient Follow Up Visits**

Follow up visits will be scheduled according to standard clinical practice, with study specific follow up visit at 6 months. This visit may be conducted at the clinical site or at the patient's home. No additional biological samples will be collected at these time points as part of this study and only clinical status regarding VL / PKDL will be assessed. If PKDL is suspected, a further skin biopsy may be taken for diagnostic confirmation.

**Identification and treatment of PKDL cases.**

The presence or absence of PKDL will be evaluated using routine diagnostic criteria in patients attending the study-specific 6 month follow up visit. If PKDL is detected, then standard care will be provided. Each site/country will avail the SOP for the standard of care.

Table 1.0. Schedule of assessments and follow-up activities

| | Clinical Assessment and consent | Confirmed diagnosis of VL | Treatment for VL | Post treatment samples | Study follow up |
|---|---|---|---|---|---|
| Visit / Observation Number | 1 | | | 2 | 3 |
| Timeline (Days) | day -2 | day -1 | day 0- day 17 | day 17 | Day180 |
| Window | +/- 2 day | +/- 1 day | | + 1-3 days | -14 days to +30 days |
| Rk39 | X | | | | |
| Diagnostic splenic / BM / LN aspirate | X* | | | | |
| Medical History | X | | | X | X |
| General Examination, vital signs | X | | X | X | X |
| Height and weight | X | | | X | X |
| PKDL examination, grading & recording | X (where applicable) | | | X where applicable | X |
| Issue information sheet and / or discuss study | X | | | | |
| Informed Consent | X | | | | |
| Skin biopsy | | X where consented | | X where consented | |
| Examination of biopsy site | | | | X | X |
| Urinalysis | X | | | | |
| Urinary pregnancy test (post-menarche females only) | X | | | | |
| Haematology & Biochemistry (5ml) | X | | | X | |
| HIV / HBV / HCV Screen(2.5ml) | X | | | | |
| Malaria screen (0.5ml) | X | | | | |
| Transcriptomic blood (2.5ml) | | X | | X | |
| Whole blood Cellular Responses (2.5ml) | | X | | X | |
| Plasma/ PBMC (10ml) | | X | | X | |
| | | | | | |
| | | | | | |
| Study Blood Volume Per Visit | 8ml | 15ml | 0ml | 20ml | 0ml |

*Residual material from diagnostic biopsies will be used for parasite culture / DNA isolation and/or RNA-Seq.

### 6.3 Additional Visits

Additional visits and assessments may be required to evaluate any adverse event and/or to clarify a diagnosis. A spleen / bone marrow or LN aspirate will be necessary to confirm diagnosis of VL prior to treatment. These visits and assessments are compatible with the protocol.

## 7.0 WITHDRAWAL, PREGNANCY, MONITORING, AND STUDY TERMINATION

### 7.1 Withdrawal of Volunteers

A volunteer has the right to withdraw from the study at any time and for any reason and is not obliged to give his or her reasons for doing so. A Clinical Investigator may withdraw the volunteer at any time in the interests of the volunteer's health and well-being. If a volunteer is considered to have failed the screening assessment or withdraws from the study at any time, either by choice or on the recommendation of clinical personnel, data and samples collected up to that point will remain available for analysis as part of the study.

If a volunteer who has withdrawn from the study requests for their existing, un-analysed samples to be destroyed or for their data to not be included in reports/ statistical analyses, the CI will take responsibility for ensuring that appropriate action is taken.

### 7.2 Pregnancy

If a female volunteer becomes pregnant during the study, she will be followed up as other volunteers.

### 7.3 Monitoring

Monitoring will be conducted by a clinical study group (CSG) comprising the Sponsor's clinical advisor and the chief investigators at each site. The first CSG meeting will be before the study starts to agree to the Terms of Reference and their schedule of meetings.

The second CSG meeting will review study data from each study site after 10 patients have been recruited at each site.

### 7.4 Sponsor

The Sponsor reserves the right to terminate the study at any time.

## 8.0 STATISTICS

### 8.1 Sample Size

This is an observational study and for many techniques, prior data is unavailable for formal power calculations. For whole blood transcriptomics, we estimate that with an sample size of 20 we would have 80% power to detect a prognostic signature of 600 genes (FDR 0.05; 2-fold change in

expression; dispersion 0.1; average read count 5) comparing between two clinical sites (https://cqs-vumc.shinyapps.io/rnaseqsamplesizeweb/). Power will be increased in paired analysis conducted on pre- vs. post-treatment samples [35]. For flow cytometry data, based on published data on the distribution of immune subset markers on PBMC from VL patients (e.g. [36]), we will need between 5 and 8 patients to detect a 50% change in the frequency of cells expressing most markers (power 80%; p=0.05). Our pilot data with tissue based Nanostring-targeted transcriptomics indicates that significant differences can be identified between pre- and post-treatment CL patients with 8 paired samples (Dey et al, unpublished). Hence, the target recruitment of 40 patients per site allows for losses to follow up, potential absence of biopsies samples from children and adolescents and occasional sample failure leading to loss of paired samples.

**8.2 Statistical Analysis Plan (SAP)**
An SAP will be produced by the study statistician before any analysis of the study data.

**9.0 MANAGEMENT OF DATA, SAMPLES AND TRIAL PROCEDURES**

**9.1 Source Data and Case Report Forms (CRFs)**
Consenting patients will be allocated a CRF, and the hospital notes will also act as source data. Successive patients screened for the study will be allocated successive Site Identification Numbers (Site ID number), e.g. G001, G002, etc. G001 etc. will be used for the Gondar site, K001 etc for Khartoum, A001 etc for Amudat, and B001 etc (B=Baringo County) for the Kenya sites. The Screening Log will hold personal identifiable information about the patient, including name, address, date of birth and Site Identification number. The patient's CRF will be held at the clinical site in a secure location. Permission will be obtained as part of the informed consent process to allow the research team and other responsible individuals access to the patient's trial records.

Data collected from the patient or from medical examinations will be entered directly into the CRF. All laboratory reports will be filed in the CRF after review and sign off by a medically qualified Clinical Investigator. Data collected at the clinical site will be transcribed directly into the Case Report Form. The type of data to be recorded in the CRF will be in line with the details provided in the study schedule section. Appendix 1 provides details of what constitutes source data in this study. CRF's will be identified with Site ID number and initials. No personal identifiable information will be sent outside of the hospital.

An appointed data manager will undertake data management responsibilities as delegated by the University of York, which include the provision of study database, data entry and validation procedures. The data manager will work with the trial team to draft the CRFs. Appropriately trained staff (named on a delegation log) will complete and send CRFs to the data manager for entry into the study database.

**9.2a Screening Log**
A screening log containing hospital numbers, name, date of birth and whether the volunteer was enrolled into the dosing part of the study will be kept in the trial site file, which will be kept in a secure location at the study site, with access restricted to study staff and monitors only.

**9.2b Volunteer Log**

A volunteer log containing hospital numbers, study number, name, date of birth and whether the volunteer was enrolled into the study will be kept securely in the study site file, with access restricted to study staff and monitors only.

**9.3 Access to Data**

The investigators will maintain appropriate medical and research records for this study. The Chief Investigator, co-investigators and clinical research nurses will have access to records. The investigators will permit authorised representatives of the sponsor(s), and regulatory agencies to examine (and when required by applicable law, to copy) clinical records for the purposes of quality assurance reviews, audits and evaluation of the study safety and progress.

**9.4 Data Protection**

The study protocol, documentation, data and all other information generated will be held in strict confidence. No information concerning the study or the data will be released to any unauthorised third party, without prior written approval of the sponsor.

**9.5 Archiving of Data**

All study documents will be securely stored for a minimum of 15 years after the close of the study in accordance with SOP on Archiving of Research Study Documents.

**9.6 Confidentiality**

Volunteers will be identified only by their study volunteer number and date of birth on any documentation or samples that leave the trial site. No personal identifiable data will be stored with external organisations. Volunteers will not be identifiable in any study report or publication.

**9.7 Management of Biological Samples**

Blood samples taken at the screening visit for routine laboratory parameters and blood borne viruses as well as routine safety parameters during the study will be tested at the local study site. University of Gondar, Gondar, Ethiopia; Kimalel and Chemolingot Health Centres Baringo County, Kenya; Professor El-Hassan Centre for Tropical Medicine, Dooka, Gedaref State, Sudan; and in Amudat Hospital, Amudat, Uganda. These samples will be identified by the volunteer’s trial number initials and date of sampling as required by the laboratory.

Due to the deteriorating security situation from civil unrest/civil war in Ethiopia and Sudan, and the associated difficulties with on-site sample analysis, research samples from all sites will instead be transported under MTAs to the KEMRI site for analyses. These will include PBMCs, plasma, skin biopsies and whole blood. All relevant laboratory staff and students from each study centre will undertake the experimental work at the KEMRI campus.

Any biological samples requiring shipping to University of York (e.g. for specialised analysis not available in country) will be subject to anMTA.

**9.8 End of the Study**

The end of the study is defined as the last visit of the last patient. The Chief Investigator will notify the national regulatory agency of the end of the trial within 90 days of its completion or within 15 days if the study is terminated early.

**9.9 Risk Assessment**

A full trial risk assessment will be carried out in accordance with the Sponsor's SOP.

**9.10 Study Monitoring**
The conduct of the trial will be monitored by the Clinical Study Group. The monitoring plan will be approved by the Research Steering Group (comprising members of the clinical and research teams).

**9.11 Trial Management**
The Sponsor delegates certain roles and responsibilities to the CI and others, as detailed in the SOP entitled "Delegation of Tasks for ImmSat@Cure".

The study will be managed by a Research Steering Group (RSG), comprising the project scientific lead, the clinical chief investigator at each site, the Sponsor's clinical representative, the study statistician, the Study Manager and the PREV_PKDL project coordinator's representative. The RSG will report to the Sponsor via the Sponsor's Clinical Trials Steering Committee, and to the funder via the PREV-PKDL management structure. The RSG will also review and comment on development of SOPs for all experimental and clinical work and the development of Case Record Files (CRFs) and Study Master File (SMF).

A Clinical Study Group (CSG) comprising Sponsor's clinical advisor and the chief investigator at each site (Professor Charles Lacey and Professor Asrat Hailu Mekuria, Dr Jane Mbui and Dr Margaret Mbuchi, Professor Ahmed Mudawi Musa, Professor Joseph Olobo, and Professor Paul Kaye), will review ongoing safety data and decide on continued recruitment. The decision of the CSG will be communicated to the RSG.

**10.0 ETHICS and ethical considerations**

**10.1 Declaration of Helsinki**
The Investigators will ensure that this study is conducted according to the principles of the current revision of the Declaration of Helsinki 2008.

**10.2 ICH Guidelines for Good Clinical Practice**
The Investigators will ensure that this study is conducted in full conformity with relevant regulations and with the ICH guidelines for GCP (CPMP/ICH/135/95) July 1996.

**10.3 Research Ethics Committee (REC)**
A copy of the protocol, informed consent forms, any other written volunteer information and the advertising material will be submitted to institutional Ethics Committees: i) Biology Ethics Committee, on behalf of the University of York Ethics Committee ii) the Scientific Review Committee at the Centre for Clinical Research, the KEMRI Scientific and Ethical Review Unit; iii) the University of Khartoum Ethics Committee; iv) the University of Makerere School of Biomedical Sciences Higher Degrees and Research Ethics Committee and v) the University of Gondar Ethics Review Committee and the Ethiopian National Research Ethics Review Committee for approval.

## 10.4 Volunteer Confidentiality

No material will be kept on file that refers to the study volunteer by their full name other than in source documentation kept at the study sites. The confidentiality of volunteers will be respected and maintained at all times.

CRFs, associated database records and blood samples will be identified by the volunteer's study number and date of birth. Study reports will contain only the volunteer's study number.

## 10.5 Regulatory and ethical approvals

This study will be conducted in accordance with the protocol and with the following: (i) Consensus ethical principles derived from international guidelines including the Declaration of Helsinki. (ii) Applicable ICH Good Clinical Practice (GCP) Guidelines. (iii) Applicable laws and regulations.
The protocol, ICF and other relevant documents will be submitted to an IRB/IEC (see 11.1, below) by the investigator and reviewed and approved by the IRB/IEC before the study is initiated. No patients or healthy controls will be recruited in the study until favourable ethics opinions have been obtained. Any amendments to the protocol will require IRB/IEC approval before implementation of changes made to the study design, except for changes necessary to eliminate an immediate hazard to study participants.

## 10.6 Informed Consent Form

The investigator or his/her representative will explain the nature of the study to the participant or his/her legally authorized representative and answer all questions regarding the study. Study information documents and ICFs will be developed in the participants' language as required by the corresponding IRB/IEC.

Participants will be informed that their participation is voluntary. Participants or their legally authorized representative (defined as an individual or juridical or other body authorized under applicable law to consent, on behalf of a prospective subject, to the subject's participation in the clinical trial) will be required to sign and date a statement of informed consent that meets the requirements of GCP ICH E6 (R2) where applicable, and the IRB/IEC or study centre.

There must be evidence that written informed consent was obtained before the participant was recruited in the study and ample time was given to participants to consent. The date and time the written consent was obtained will be recorded. The authorized person obtaining the informed consent will also sign and date the ICF.

Non-literate participants must provide a thumbprint on the ICF and the ICF signed and dated by an impartial witness (who is independent of the trial, who cannot be unfairly influenced by people involved with the trial, who attends the informed consent process if the subject or the subject's legally acceptable representative cannot read, and who reads the informed consent form and any other written information supplied to the subject).

For minors (< 18 years old) participating in the study, parental consent will be sought from the legally authorized representative. Assent will be obtained for children aged 9 – 11 and adolescents aged

12-17 years old in addition to parental consent. However, in all children, any opposition, resistance or protest to study procedures will be discussed with the parents/legally designated representative to analyse whether the behaviour is merely an expression of the anticipated but acceptable burden, or is reason for concern on research continuation. When the analysis concludes that these are expressions of dissent, it would be respected.

In the case of an amendment to the study protocol, participants will be re-consented to the most current version of the ICF(s) during their participation in the study. A signed copy of the ICF(s) will be provided to the participant or the participant's legally authorized representative.

**10.7 Risks**

The Investigators will ensure that the dignity, rights, safety and well-being of volunteers are given priority at all times.

There is a small risk of pain and discomfort while taking blood and performing the skin biopsy. All efforts will be taken to minimise pain from the biopsy by using a local anaesthesia, and providing anti-inflammatory drugs to the patient after the procedure. The procedure will also be done using antiseptic techniques to minimize any probability of infection at biopsy site. A minimal scar is expected and patients advised of this during consent. Assessment of the healing of the first biopsy will be carried out prior to conducting a second biopsy. Patients will be able to remain in the study without consenting to the skin biopsy.

All patient procedures related to their treatment for VL will be carried out in accordance with local clinical guidelines

**10.8 Benefits**

There are no direct benefits envisaged to volunteers in this study, but the benefits of participating will be explained (new understanding of disease leading to better diagnostic, preventative measures or treatment options in the future).

Volunteers will receive a thorough medical examination. In the event of any abnormal findings, volunteers will be advised of the best course of action and referred on to the appropriate clinician where appropriate.

**10.9 HIV Testing**

Volunteers will be required to undergo HIV testing as part of the eligibility screening assessment. Volunteers will receive pre-test counselling in line with current local practice.

**10.10 Reimbursement**

Volunteers will be compensated for their time and for any costs incurred to them by participating in the study (e.g. transport costs). Details of compensation are provided in section 13 and in the informed consent form.

## 11.0 REGULATORY AND GOVERNANCE ISSUES

This is an observational study only and no investigational medicinal products are being evaluated.

### 11.1 Required approvals

Current regulatory frameworks for this study require that the following approvals and registration must be obtained before commencement of the study:

- Clintrials.gov Registration
- Ethical approval from Institutional Ethics Committees (University of Khartoum, KEMRI, University of Gondar, Makerere University)
- Ethical Approval from University of York, UK
- Ethical approval from National Ethics Committee (for material transfer: Ethiopian National Research Ethics Review Committee)

### 11.2 Amendments

Both substantial and non-substantial amendments will be submitted according to the Sponsor's recommendations for submitting amendments.

### 11.2 GCP, and GLP Compliance

All clinical staff will receive GCP training. The study will be monitored and audited in line with GCP requirements.

## 12.0 INDEMNITY

The University of York will act as Sponsor of the clinical study and holds insurance for claims from study participants for harm for which it is legally liable arising from its design or management of the clinical study.

### 12.1 Negligent Harm

The University of York will provide indemnity and compensation in the event of a claim by, or on behalf of participants, for harm as a result of the Sponsor's negligence in the design or management of the clinical study.

## 13.0 FINANCE

### 13.1 Financing

The study will be funded by an EDCTP grant (REFERENCE: RIA2016V-1640)

### 13.2 Reimbursement for Volunteers

Volunteers will be compensated for their time and for the inconvenience caused by procedures at the following visits:

6 month follow up visit (as per local site specific protocol)

Treatment and investigations associated with the patient's diagnosis of VL and or their treatment will be provided free of charge for those who need it.

Healthy volunteers will be compensated at local rates for their time and receive a free health assessment from one of the study physicians as well having routine haematology and biochemistry tests checked.

## 14.0 PUBLICATION

The results of the study will be analysed and prepared in a study report for publication in a peer reviewed professional journal. The Chief Investigators, Professor Charles Lacey, the Statistical Investigator and associated researchers will form the basis of the writing group. Authorship will reflect work done by the Investigators.

## 15.0 TRAINING AND CAPACITY BUILDING

The study will employ local early career researchers and clinical staff. These will receive training in clinical studies, GCP, advanced immunology and pathology techniques and data analysis. This study forms part of a larger capacity building grant from EDCTP that will develop an EAR centre of excellence in flow cytometry, provision of new instrumentation in each country and access to national and international training courses, managed by University of York.

## 16.0 REFERENCES


1. Alvar J, Velez ID, Bern C, Herrero M, Desjeux P, Cano J, et al. Leishmaniasis worldwide and global estimates of its incidence. PloS one. 2012;7(5):e35671. Epub 2012/06/14. doi: 10.1371/journal.pone.0035671. PubMed PMID: 22693548; PubMed Central PMCID: PMC3365071.
2. Musa AM, Younis B, Fadlalla A, Royce C, Balasegaram M, Wasunna M, et al. Paromomycin for the treatment of visceral leishmaniasis in Sudan: a randomized, open-label, dose-finding study. PLoS neglected tropical diseases. 2010;4(10):e855. Epub 2010/11/05. doi: 10.1371/journal.pntd.0000855. PubMed PMID: 21049063; PubMed Central PMCID: PMC2964291.
3. Mueller M, Ritmeijer K, Balasegaram M, Koummuki Y, Santana MR, Davidson R. Unresponsiveness to AmBisome in some Sudanese patients with kala-azar. Transactions of the Royal Society of Tropical Medicine and Hygiene. 2007;101(1):19-24. Epub 2006/05/30. doi: 10.1016/j.trstmh.2006.02.005. PubMed PMID: 16730363.
4. Adriaensen W, Dorlo TPC, Vanham G, Kestens L, Kaye PM, van Griensven J. Immunomodulatory Therapy of Visceral Leishmaniasis in Human Immunodeficiency Virus-Coinfected Patients. Frontiers in immunology. 2017;8:1943. Epub 2018/01/30. doi: 10.3389/fimmu.2017.01943. PubMed PMID: 29375567; PubMed Central PMCID: PMCPMC5770372.
5. Khalil EA, Khidir SA, Musa AM, Musa BY, Elfaki ME, Elkadaru AM, et al. Post-Kala-Azar Dermal Leishmaniasis: A Paradigm of Paradoxical Immune Reconstitution Syndrome in Non-HIV/AIDS Patients. Journal of tropical medicine. 2013;2013:275253. Epub 2013/05/02. doi: 10.1155/2013/275253. PubMed PMID: 23634148; PubMed Central PMCID: PMC3619621.
6. Mukhopadhyay D, Dalton JE, Kaye PM, Chatterjee M. Post kala-azar dermal leishmaniasis: an unresolved mystery. Trends in parasitology. 2014;30(2):65-74. Epub 2014/01/07. doi: 10.1016/j.pt.2013.12.004. PubMed PMID: 24388776; PubMed Central PMCID: PMC3919212.
7. Zijlstra EE. PKDL and other dermal lesions in HIV co-infected patients with Leishmaniasis: review of clinical presentation in relation to immune responses. PLoS neglected tropical diseases. 2014;8(11):e3258. Epub 2014/11/21. doi: 10.1371/journal.pntd.0003258. PubMed PMID: 25412435; PubMed Central PMCID: PMC4238984.
8. Zijlstra EE. The immunology of post-kala-azar dermal leishmaniasis (PKDL). Parasites & vectors. 2016;9:464. Epub 2016/08/25. doi: 10.1186/s13071-016-1721-0. PubMed PMID: 27553063; PubMed Central PMCID: PMC4995613.
9. Desjeux P, Ghosh RS, Dhalaria P, Strub-Wourgaft N, Zijlstra EE. Report of the Post Kala-azar Dermal Leishmaniasis (PKDL) Consortium Meeting, New Delhi, India, 27-29 June 2012. Parasites & vectors. 2013;6:196. Epub 2013/07/04. doi: 10.1186/1756-3305-6-196. PubMed PMID: 23819611; PubMed Central PMCID: PMC3733610.
10. Ganguly S, Das NK, Barbhuiya JN, Chatterjee M. Post-kala-azar dermal leishmaniasis--an overview. International journal of dermatology. 2010;49(8):921-31. Epub 2010/12/07. doi: 10.1111/j.1365-4632.2010.04558.x. PubMed PMID: 21128917.
11. Mondal D, Bern C, Ghosh D, Rashid M, Molina R, Chowdhury R, et al. Quantifying the infectiousness of post-kala-azar dermal leishmaniasis towards sandflies. Clinical infectious diseases : an official publication of the Infectious Diseases Society of America. 2018. Epub 2018/10/26. doi: 10.1093/cid/ciy891. PubMed PMID: 30357373.
12. Zijlstra EE, Alves F, Rijal S, Arana B, Alvar J. Post-kala-azar dermal leishmaniasis in the Indian subcontinent: A threat to the South-East Asia Region Kala-azar Elimination Programme. PLoS neglected tropical diseases. 2017;11(11):e0005877. Epub 2017/11/18. doi: 10.1371/journal.pntd.0005877. PubMed PMID: 29145397; PubMed Central PMCID: PMCPMC5689828.
13. Hamad SH, Khalil EA, Musa AM, Ibrahim ME, Younis BM, Elfaki ME, et al. Leishmania donovani: genetic diversity of isolates from Sudan characterized by PCR-based RAPD.

Experimental parasitology. 2010;125(4):389-93. Epub 2010/03/30. doi: 10.1016/j.exppara.2010.03.008. PubMed PMID: 20346944.
14. Khalil EA, Weldegebreal T, Younis BM, Omollo R, Musa AM, Hailu W, et al. Safety and efficacy of single dose versus multiple doses of AmBisome for treatment of visceral leishmaniasis in eastern Africa: a randomised trial. PLoS neglected tropical diseases. 2014;8(1):e2613. Epub 2014/01/24. doi: 10.1371/journal.pntd.0002613. PubMed PMID: 24454970; PubMed Central PMCID: PMC3894173.
15. Berman JD, Badaro R, Thakur CP, Wasunna KM, Behbehani K, Davidson R, et al. Efficacy and safety of liposomal amphotericin B (AmBisome) for visceral leishmaniasis in endemic developing countries. Bull World Health Organ. 1998;76(1):25-32. Epub 1998/06/06. PubMed PMID: 9615494; PubMed Central PMCID: PMCPMC2305623.
16. Musa AM, Khalil EA, Younis BM, Elfaki ME, Elamin MY, Adam AO, et al. Treatment-based strategy for the management of post-kala-azar dermal leishmaniasis patients in the Sudan. Journal of tropical medicine. 2013;2013:708391. Epub 2013/05/22. doi: 10.1155/2013/708391. PubMed PMID: 23690794; PubMed Central PMCID: PMC3649346.
17. Imamura H, Downing T, Van den Broeck F, Sanders MJ, Rijal S, Sundar S, et al. Evolutionary genomics of epidemic visceral leishmaniasis in the Indian subcontinent. eLife. 2016;5. Epub 2016/03/24. doi: 10.7554/eLife.12613. PubMed PMID: 27003289; PubMed Central PMCID: PMC4811772.
18. Nylen S, Maurya R, Eidsmo L, Manandhar KD, Sundar S, Sacks D. Splenic accumulation of IL-10 mRNA in T cells distinct from CD4+CD25+ (Foxp3) regulatory T cells in human visceral leishmaniasis. The Journal of experimental medicine. 2007;204(4):805-17. Epub 2007/03/29. doi: 10.1084/jem.20061141. PubMed PMID: 17389235; PubMed Central PMCID: PMC2118563.
19. Faleiro RJ, Kumar R, Hafner LM, Engwerda CR. Immune regulation during chronic visceral leishmaniasis. PLoS neglected tropical diseases. 2014;8(7):e2914. Epub 2014/07/11. doi: 10.1371/journal.pntd.0002914. PubMed PMID: 25010815; PubMed Central PMCID: PMCPMC4091888.
20. Gautam S, Kumar R, Maurya R, Nylen S, Ansari N, Rai M, et al. IL-10 neutralization promotes parasite clearance in splenic aspirate cells from patients with visceral leishmaniasis. The Journal of infectious diseases. 2011;204(7):1134-7. Epub 2011/09/02. doi: 10.1093/infdis/jir461. PubMed PMID: 21881130; PubMed Central PMCID: PMC3164427.
21. Kumar R, Chauhan SB, Ng SS, Sundar S, Engwerda CR. Immune Checkpoint Targets for Host-Directed Therapy to Prevent and Treat Leishmaniasis. Frontiers in immunology. 2017;8:1492. Epub 2017/11/24. doi: 10.3389/fimmu.2017.01492. PubMed PMID: 29167671; PubMed Central PMCID: PMCPMC5682306.
22. Gautam S, Kumar R, Singh N, Singh AK, Rai M, Sacks D, et al. CD8 T cell exhaustion in human visceral leishmaniasis. The Journal of infectious diseases. 2014;209(2):290-9. Epub 2013/08/08. doi: 10.1093/infdis/jit401. PubMed PMID: 23922369; PubMed Central PMCID: PMCPMC3873784.
23. Binnewies M, Roberts EW, Kersten K, Chan V, Fearon DF, Merad M, et al. Understanding the tumor immune microenvironment (TIME) for effective therapy. Nature medicine. 2018;24(5):541-50. Epub 2018/04/25. doi: 10.1038/s41591-018-0014-x. PubMed PMID: 29686425; PubMed Central PMCID: PMCPMC5998822.
24. Chauhan SB, Faleiro R, Kumar R, Ng S, Singh B, Singh OP, et al. IL-2 is an upstream regulator of CD4+ T cells from visceral leishmaniasis patients with therapeutic potential. The Journal of infectious diseases. 2019. Epub 2019/02/24. doi: 10.1093/infdis/jiz074. PubMed PMID: 30796820.
25. Gardinassi LG, Garcia GR, Costa CH, Costa Silva V, de Miranda Santos IK. Blood Transcriptional Profiling Reveals Immunological Signatures of Distinct States of Infection of Humans with Leishmania infantum. PLoS neglected tropical diseases. 2016;10(11):e0005123. Epub 2016/11/10. doi: 10.1371/journal.pntd.0005123. PubMed PMID: 27828962; PubMed Central PMCID: PMCPMC5102635.

26. Salih MAM, Fakiola M, Lyons PA, Younis BM, Musa AM, Elhassan AM, et al. Expression profiling of Sudanese visceral leishmaniasis patients pre- and post-treatment with sodium stibogluconate. Parasite immunology. 2017;39(6). Epub 2017/04/04. doi: 10.1111/pim.12431. PubMed PMID: 28370072.
27. Mukherjee S, Sengupta R, Mukhopadhyay D, Braun C, Mitra S, Roy S, et al. Impaired activation of lesional CD8(+) T-cells is associated with enhanced expression of Programmed Death-1 in Indian Post Kala-azar Dermal Leishmaniasis. Scientific reports. 2019;9(1):762. Epub 2019/01/27. doi: 10.1038/s41598-018-37144-y. PubMed PMID: 30679687; PubMed Central PMCID: PMCPMC6345993.
28. Mukhopadhyay D, Mukherjee S, Roy S, Dalton JE, Kundu S, Sarkar A, et al. M2 Polarization of Monocytes-Macrophages Is a Hallmark of Indian Post Kala-Azar Dermal Leishmaniasis. PLoS neglected tropical diseases. 2015;9(10):e0004145. Epub 2015/10/27. doi: 10.1371/journal.pntd.0004145. PubMed PMID: 26496711; PubMed Central PMCID: PMC4619837.
29. Ismail A, Khalil EA, Musa AM, El Hassan IM, Ibrahim ME, Theander TG, et al. The pathogenesis of post kala-azar dermal leishmaniasis from the field to the molecule: does ultraviolet light (UVB) radiation play a role? Medical hypotheses. 2006;66(5):993-9. Epub 2006/01/03. doi: 10.1016/j.mehy.2005.03.035. PubMed PMID: 16386855.
30. Gasim S, Elhassan AM, Khalil EA, Ismail A, Kadaru AM, Kharazmi A, et al. High levels of plasma IL-10 and expression of IL-10 by keratinocytes during visceral leishmaniasis predict subsequent development of post-kala-azar dermal leishmaniasis. Clin Exp Immunol. 1998;111(1):64-9. Epub 1998/02/24. PubMed PMID: 9472662; PubMed Central PMCID: PMCPMC1904865.
31. Gorski S, Collin SM, Ritmeijer K, Keus K, Gatluak F, Mueller M, et al. Visceral leishmaniasis relapse in Southern Sudan (1999-2007): a retrospective study of risk factors and trends. PLoS neglected tropical diseases. 2010;4(6):e705. Epub 2010/06/15. doi: 10.1371/journal.pntd.0000705. PubMed PMID: 20544032; PubMed Central PMCID: PMC2882338.
32. Courtenay O, Carson C, Calvo-Bado L, Garcez LM, Quinnell RJ. Heterogeneities in Leishmania infantum infection: using skin parasite burdens to identify highly infectious dogs. PLoS neglected tropical diseases. 2014;8(1):e2583. Epub 2014/01/15. doi: 10.1371/journal.pntd.0002583. PubMed PMID: 24416460; PubMed Central PMCID: PMC3886905.
33. Doehl JSP, Bright Z, Dey S, Davies H, Magson J, Brown N, et al. Skin parasite landscape determines host infectiousness in visceral leishmaniasis. Nature communications. 2017;8(1):57. Epub 2017/07/07. doi: 10.1038/s41467-017-00103-8. PubMed PMID: 28680146; PubMed Central PMCID: PMCPMC5498584.
34. Kirstein OD, Abbasi I, Horwitz BZ, Skrip L, Hailu A, Jaffe C, et al. Minimally invasive microbiopsies: a novel sampling method for identifying asymptomatic, potentially infectious carriers of Leishmania donovani. International journal for parasitology. 2017;47(10-11):609-16. Epub 2017/04/30. doi: 10.1016/j.ijpara.2017.02.005. PubMed PMID: 28455239; PubMed Central PMCID: PMCPMC5596977.
35. Stevens JR, Herrick JS, Wolff RK, Slattery ML. Power in pairs: assessing the statistical value of paired samples in tests for differential expression. BMC Genomics. 2018;19(1):953. Epub 2018/12/24. doi: 10.1186/s12864-018-5236-2. PubMed PMID: 30572829; PubMed Central PMCID: PMCPMC6302489.
36. Faleiro RJ, Kumar R, Bunn PT, Singh N, Chauhan SB, Sheel M, et al. Combined Immune Therapy for the Treatment of Visceral Leishmaniasis. PLoS neglected tropical diseases. 2016;10(2):e0004415. Epub 2016/02/13. doi: 10.1371/journal.pntd.0004415. PubMed PMID: 26872334; PubMed Central PMCID: PMC4752322.

**Appendix 1: Source Data Definition**

| Type of Data | Source Document |
|---|---|
| Informed consent | Paper copy in site files |
| Relevant Medical History and Current Medical Conditions | CRF and medical notes |
| Demographics | CRF |
| Physical Examination and Observations | CRF |
| Clinical Laboratory Reports – Haematology, Biochemistry, HIV, | Printed report form, kept in CRF |
| Urinalysis, Urine pregnancy tests | CRF |
| Fulfilment of eligibility criteria | CRF |
| Date/time of immunogenicity sampling | CRF |
| Date of visits/examinations | CRF |
| Protocol Deviation | File note in Study Master File |
| Withdrawal | CRF |
