# Supplementary material for: Systemic inflammatory markers of visceral leishmaniasis treatment response in East Africa
Source: PLoS Negl Trop Dis. 2026 Feb 27;20(2):e0013749. doi: 10.1371/journal.pntd.0013749 (PMC12965683; doi:10.1371/journal.pntd.0013749)
Supplement: S1 Fig — Each row corresponds to a clinical/Haematological/Immune marker, and each column corresponds to a patient. Red cells represent missing data. The colour-strip at the bottom represents the country of origin of each sample. (DOCX) [file pntd.0013749.s004.docx]

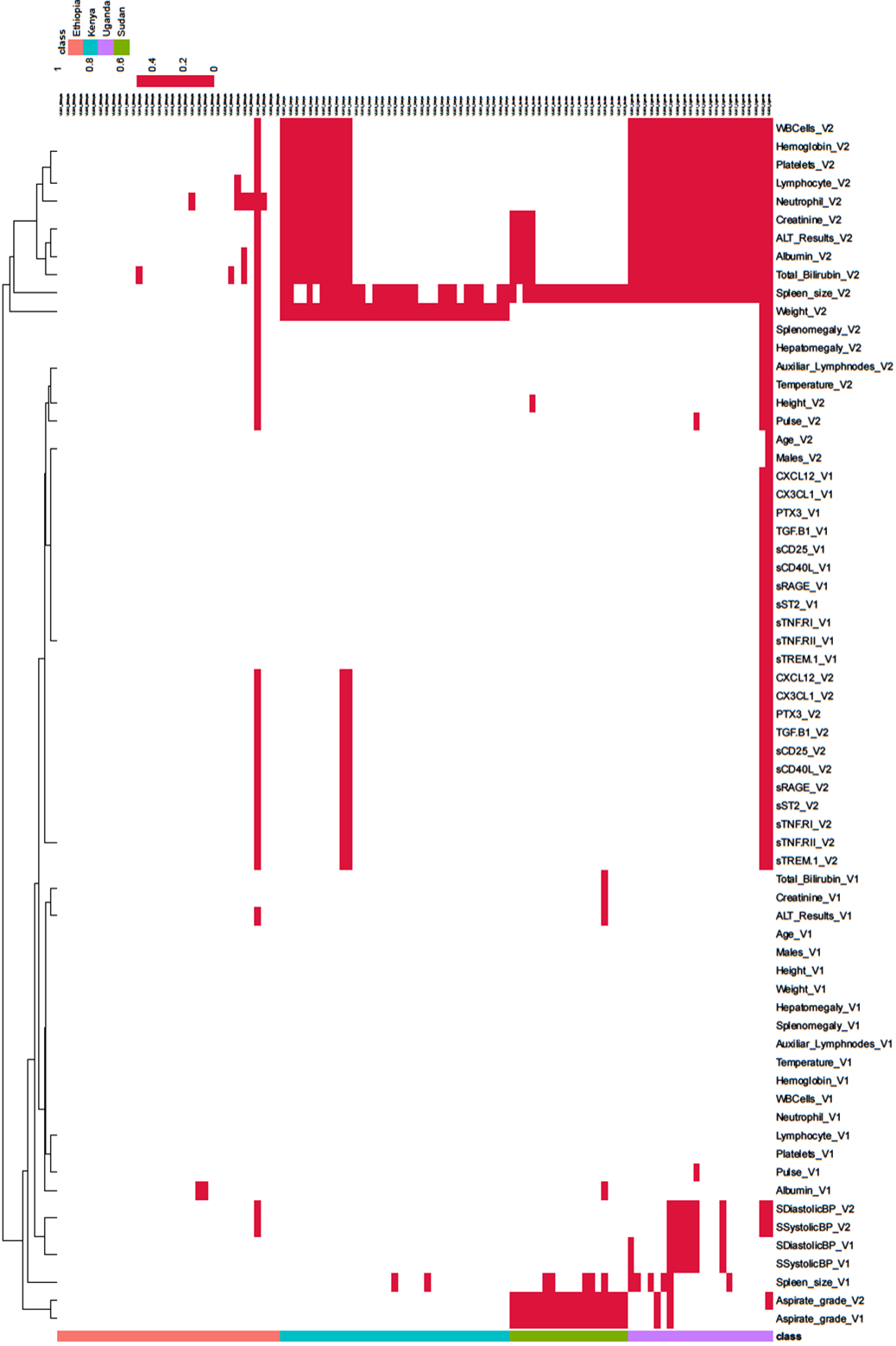


**Supplementary Figure 1: Missing data across patients.** Each row corresponds to a clinical/Hematological/Immune marker, and each column corresponds to a patient. Red cells represent missing data. The colour-strip at the bottom represents the country of origin of each sample.
