# Supplementary material for: Systemic inflammatory markers of visceral leishmaniasis treatment response in East Africa
Source: PLoS Negl Trop Dis. 2026 Feb 27;20(2):e0013749. doi: 10.1371/journal.pntd.0013749 (PMC12965683; doi:10.1371/journal.pntd.0013749)
Supplement: S2 Fig — Violin plots showing the range and statistical support for the variations in clinical, haematological and inflammation markers pre and post treatment. The numbers of asterisks represent Wilcoxon signed-rank test (V1 x V2 comparison) or Mann-Whitney U test (HV comparing to V1 or V2) p-values, where 1–4 corresponds respectively values below 0,05, 0.01, 0.001 and 0.0001. A) Males Ethiopia, B) Males Kenya, C) Males Sudan, D) Males Uganda, E) Females Kenya, F) Females Sudan, G) Females Uganda. Most of the after treatment clinical data for Uganda is missing. There is only one Female HV in Kenya. (DOCX) [file pntd.0013749.s005.docx]

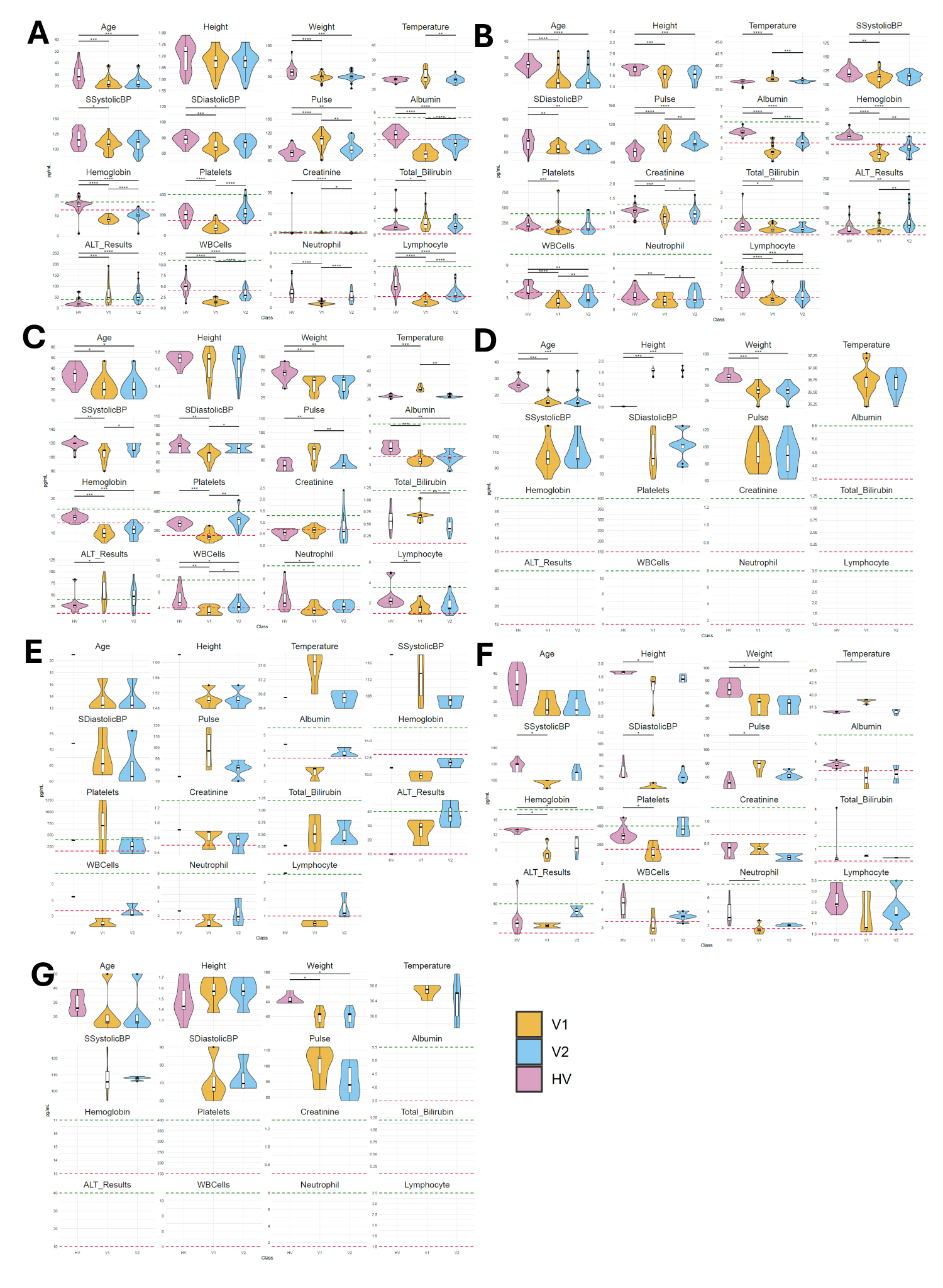


**Supplementary Figure 2: Clinical data changes with VL patient treatment.** Violin plots showing the range and statistical support for the variations in clinical, hematological and inflammation markers pre and post treatment. The numbers of asterisks represent Wilcoxon signed-rank test (V1 x V2 comparison) or Mann-Whitney U test (HV comparing to V1 or V2) p-values, where 1 to 4 corresponds respectively values below 0,05, 0.01, 0.001 and 0.0001. **A)** Males Ethiopia, **B)** Males Kenya, **C)** Males Sudan, **D)** Males Uganda, **E)** Females Kenya, **F)** Females Sudan, **G)** Females Uganda. Most of the after treatment clinical data for Uganda is missing. There is only one Female HV in Kenya.
