# Supplementary material for: Systemic inflammatory markers of visceral leishmaniasis treatment response in East Africa
Source: PLoS Negl Trop Dis. 2026 Feb 27;20(2):e0013749. doi: 10.1371/journal.pntd.0013749 (PMC12965683; doi:10.1371/journal.pntd.0013749)
Supplement: S4 Fig — Each panel corresponds to a different trait. The normal reference ranges for the traits are represented by green (upper normal limit) and red (lower normal limit) lines. Patients are identified by a combination of colour and shape. A) Females Ethiopia, B) Females Kenya, C) Females Sudan, D) Females Uganda. (DOCX) [file pntd.0013749.s007.docx]

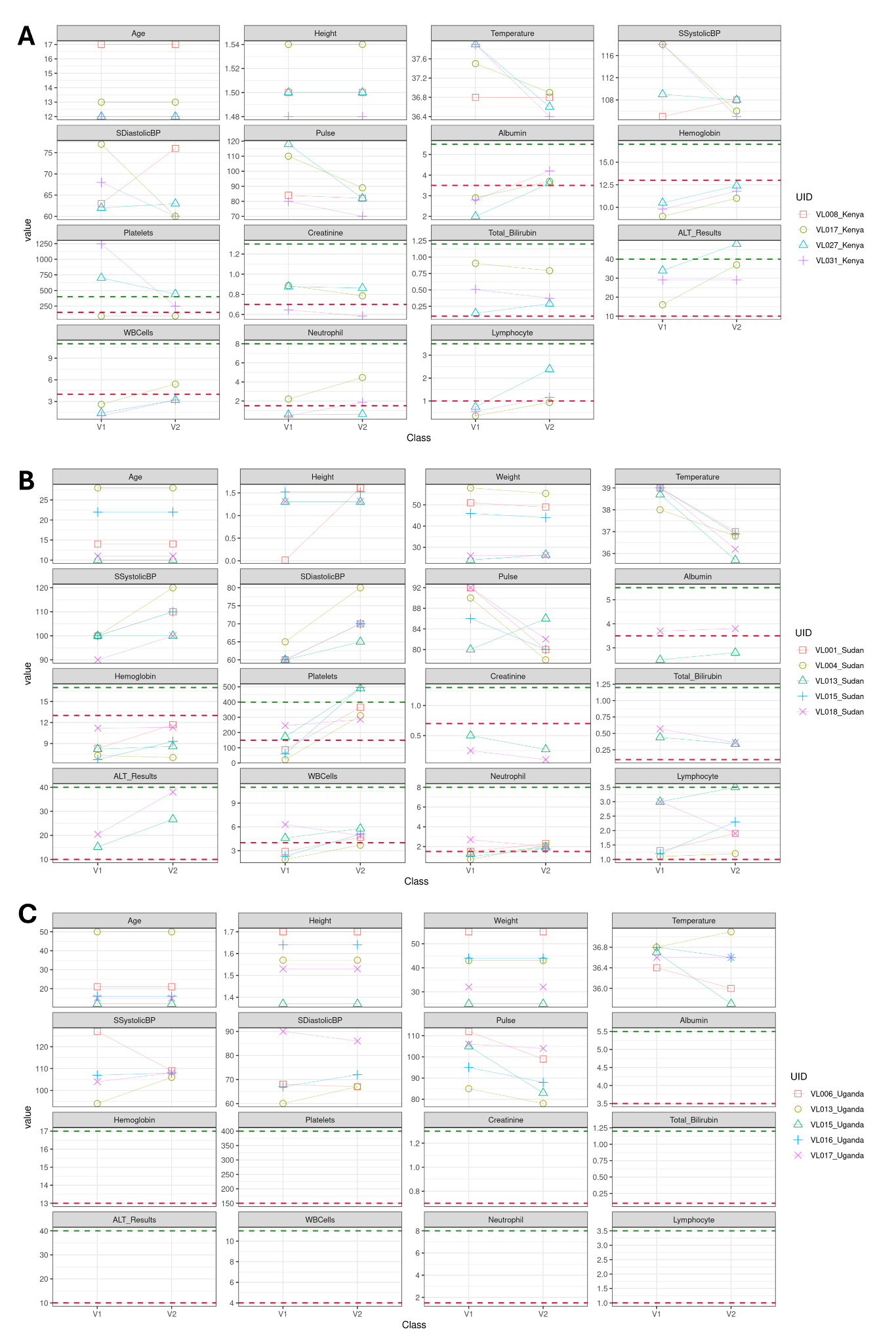


**Supplementary Figure 4: Slopegraph showing female patient’s trait levels before (V1) and after (V2) treatment.** Each panel corresponds to a different trait. The normal reference ranges for the traits are represented by green (upper normal limit) and red (lower normal limit) lines. Patients are identified by a combination of colour and shape. **A)** Females Ethiopia, **B)** Females Kenya, **C)** Females Sudan, **D)** Females Uganda.
