# Supplementary material for: Systemic inflammatory markers of visceral leishmaniasis treatment response in East Africa
Source: PLoS Negl Trop Dis. 2026 Feb 27;20(2):e0013749. doi: 10.1371/journal.pntd.0013749 (PMC12965683; doi:10.1371/journal.pntd.0013749)
Supplement: S5 Fig — Missing data is represented in gray (NA). Interval ranges for each patient group can be seen in S1 Table. Statistical comparisons between V1, V2 and HV can be seen in S2 Table. A) All data. B) Male patient data. C) Female patient data. D) Convenience controls data. (DOCX) [file pntd.0013749.s008.docx]

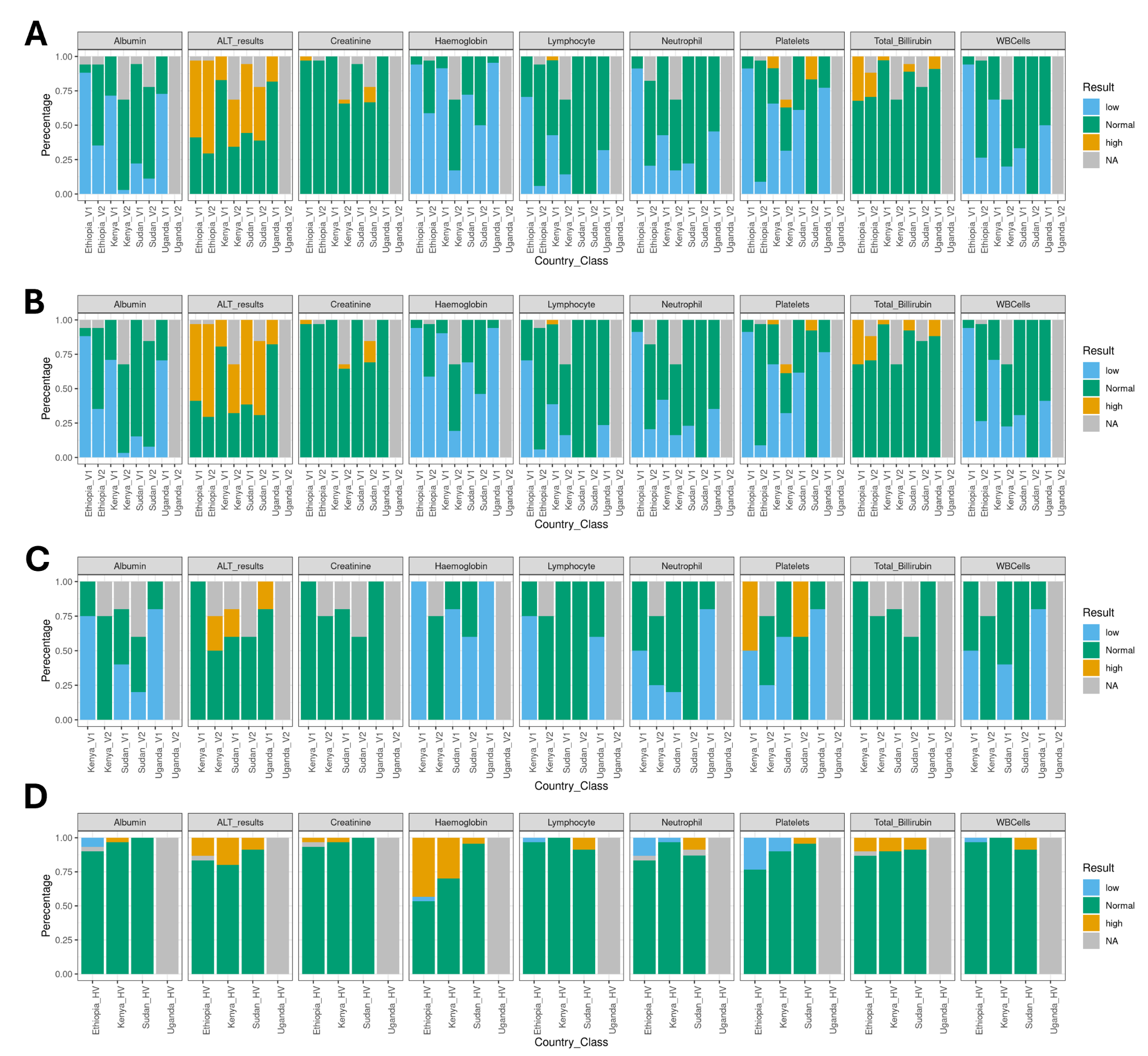


**Supplementary Figure 5:**  **Proportion of patients’ clinical traits that were below (low) within range (green) or above the normal range for each haematological marker accessed**. Missing data is represented in gray (NA). Interval ranges for each patient group can be seen in [Supplementary table 1](https://docs.google.com/spreadsheets/d/1jLhf3W7OjsqHCiBGJgfU4IYn_rQ58U1y/edit?usp=drive_link&ouid=105683387679222184223&rtpof=true&sd=true). Statistical comparisons between V1, V2 and HV can be seen in Supplementary Figure BBB and in [Supplementary table 2](https://docs.google.com/spreadsheets/d/1jLhf3W7OjsqHCiBGJgfU4IYn_rQ58U1y/edit?usp=drive_link&ouid=105683387679222184223&rtpof=true&sd=true). **A)** All data. **B)** Male patient data. **C)** Female patient data. **D)** Convenience controls data.
