# Supplementary material for: Systemic inflammatory markers of visceral leishmaniasis treatment response in East Africa
Source: PLoS Negl Trop Dis. 2026 Feb 27;20(2):e0013749. doi: 10.1371/journal.pntd.0013749 (PMC12965683; doi:10.1371/journal.pntd.0013749)
Supplement: S6 Fig — Violin plots showing the range and statistical support for the variations in clinical, haematological and inflammation markers in males (red) and females (blue). The numbers of asterisks represent Mann-Whitney U test p-values, where 1–4 corresponds respectively values below 0,05, 0.01, 0.001 and 0.0001. (DOCX) [file pntd.0013749.s009.docx]

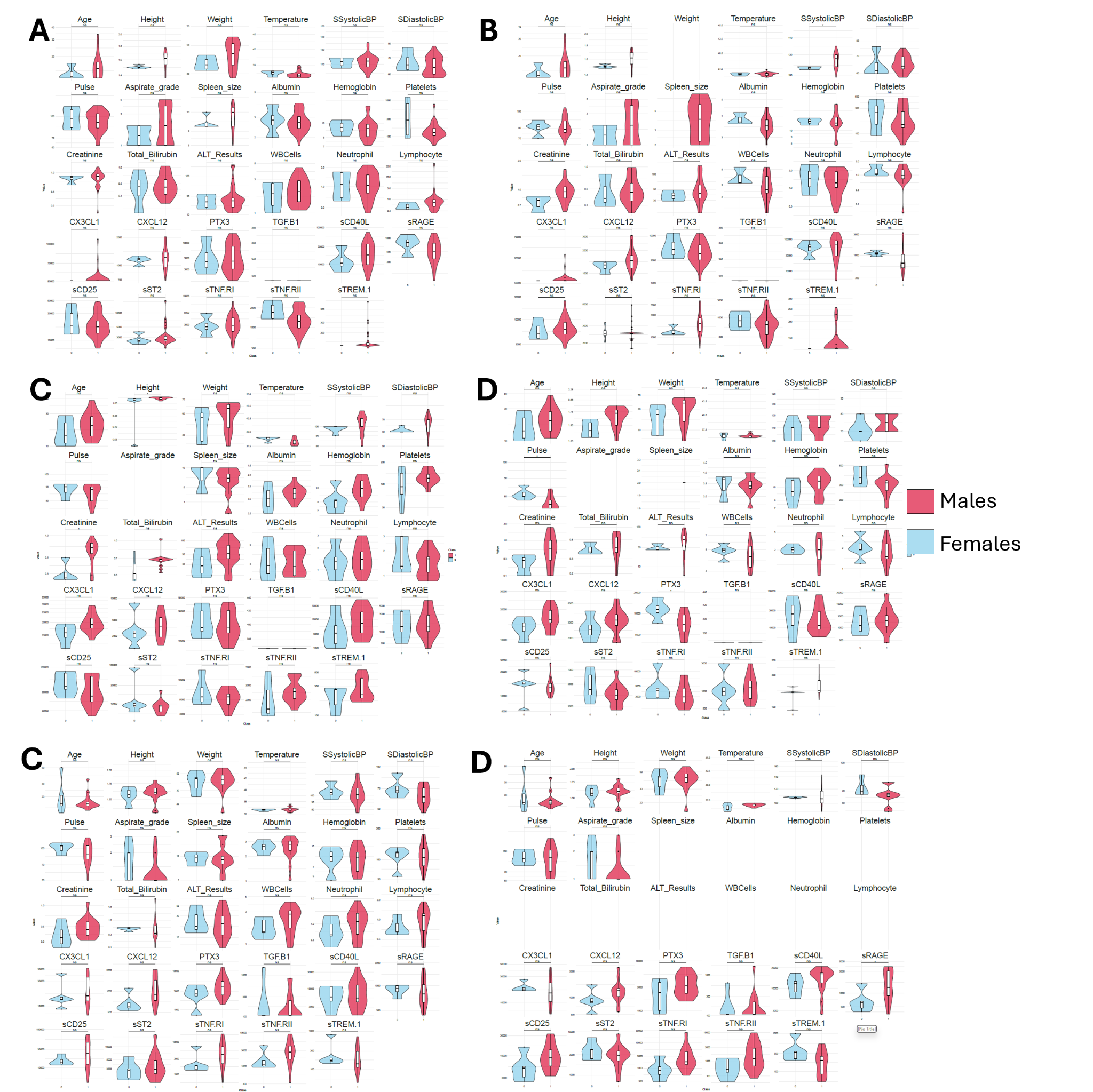


**Supplementary Figure 6: Male-Female clinical, hematological and inflammatory markers comparison.** Violin plots showing the range and statistical support for the variations in clinical, hematological and inflammation markers in males (red) and females (blue). The numbers of asterisks represent Mann-Whitney U test p-values, where 1 to 4 corresponds respectively values below 0,05, 0.01, 0.001 and 0.0001.
