# Supplementary material for: Systemic inflammatory markers of visceral leishmaniasis treatment response in East Africa
Source: PLoS Negl Trop Dis. 2026 Feb 27;20(2):e0013749. doi: 10.1371/journal.pntd.0013749 (PMC12965683; doi:10.1371/journal.pntd.0013749)
Supplement: S7 Fig — Each panel represents a different marker, and each different plate used in the experiment is shown by different colour. The red line represents the Limit of Quantification (LOQ) used for each country, which was based on the higher LOQ for a given marker for all plates from a given country. A) Ethiopia, B) Kenya, C) Sudan, D) Uganda. (DOCX) [file pntd.0013749.s010.docx]

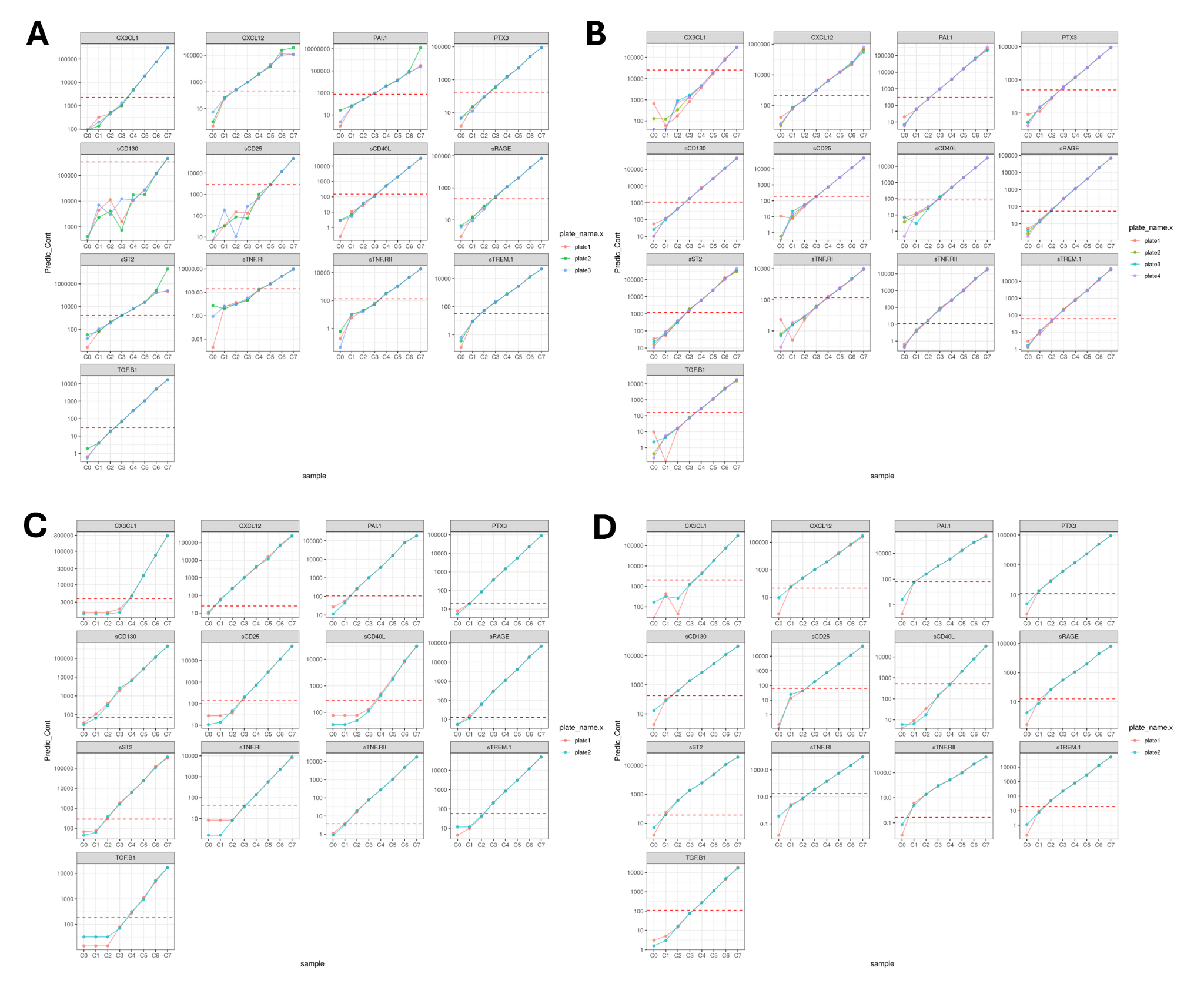


**Supplementary Figure 7: LEGENDplex standard curves.** Each panel represents a different marker, and each different plate used in the experiment is shown by different colour. The red line represents the Limit of Quantification (LOQ) used for each country, which was based on the higher LOQ for a given marker for all plates from a given country. **A)** Ethiopia, **B)** Kenya, **C)** Sudan, **D)** Uganda.
