# Supplementary material for: Systemic inflammatory markers of visceral leishmaniasis treatment response in East Africa
Source: PLoS Negl Trop Dis. 2026 Feb 27;20(2):e0013749. doi: 10.1371/journal.pntd.0013749 (PMC12965683; doi:10.1371/journal.pntd.0013749)
Supplement: S8 Fig — A) Ethiopia. B) Kenya. C) Sudan. D) Uganda. (DOCX) [file pntd.0013749.s011.docx]

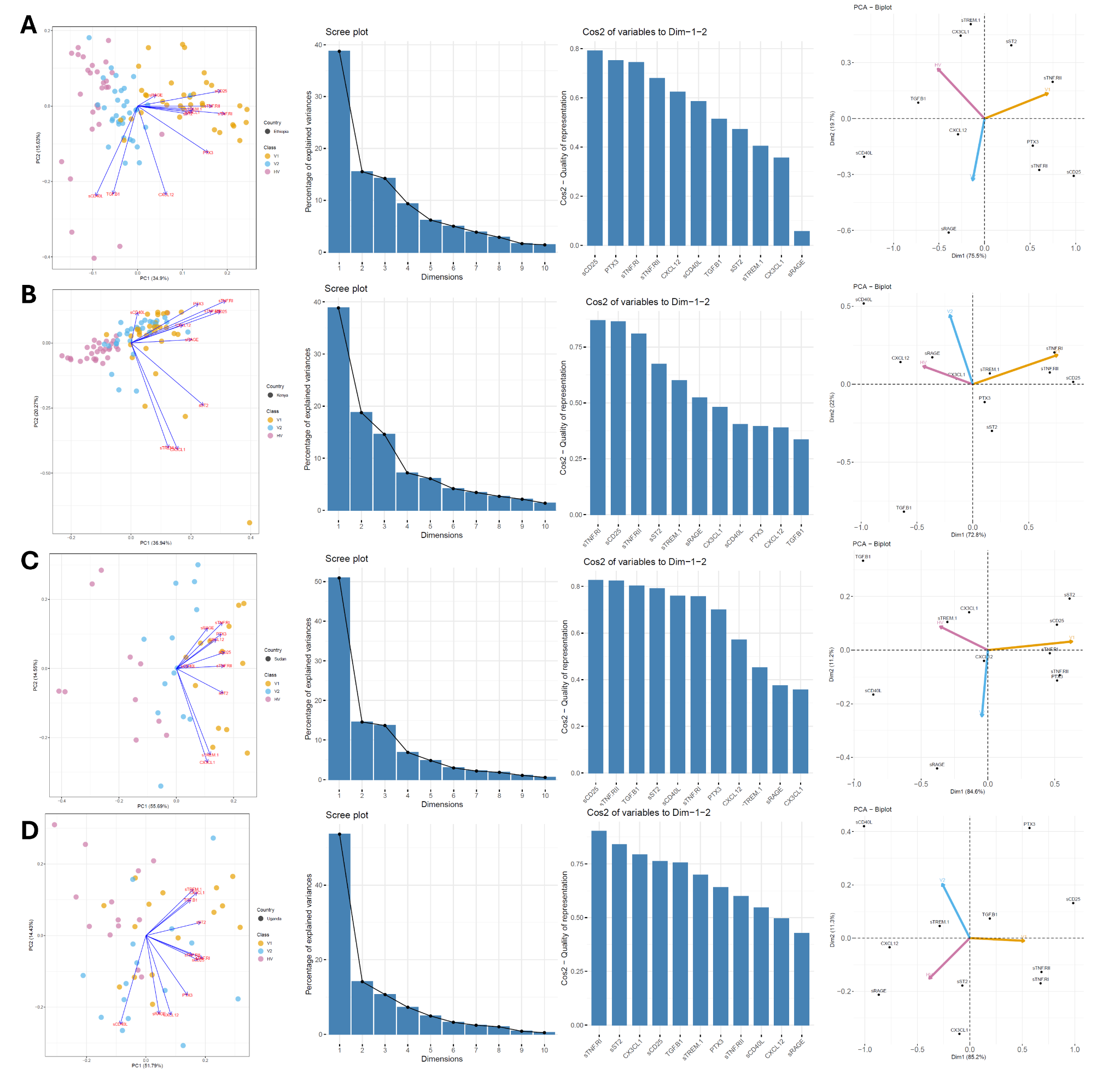


**Supplementary figure 8: PCA, Scree plot, loading plot and patient group biplot for each country. A)** Ethiopia. **B)** Kenya. **C)** Sudan. **D)** Uganda.
