# Supplementary material for: Systemic inflammatory markers of visceral leishmaniasis treatment response in East Africa
Source: PLoS Negl Trop Dis. 2026 Feb 27;20(2):e0013749. doi: 10.1371/journal.pntd.0013749 (PMC12965683; doi:10.1371/journal.pntd.0013749)
Supplement: S9 Fig — The left panel is a PCA biplot of the inflammation markers values for each patient. Each patient is represented by a dot, coloured in yellow (V1) blue (V2) and red (HV). The arrows represent how much each immune marker contributes to the principal components. The middle panel is a radar plot, where each segment corresponds to the median of patients in a group (V1, V2 or HV), scaled to the highest median in the three groups (ex: if V1, V2 and HV were respectively 20, 10 and 5, they would be represented as 100, 50 and 25) of one inflammation marker. Markers that were significantly (p < 0.05) higher in V1 or V2 are respectively coloured in yellow or blue. Markers that were not significantly different are in black. Violin plots showing the range and statistical support for the variations in inflammation markers pre and post treatment. The numbers of asterisks represent p-values, where 1–4 corresponds respectively values below 0,05, 0.01, 0.001 and 0.0001. The letters correspond to the results from A) Ethiopia, B) Kenya, C) Sudan and D) Uganda. The statistical results and number of samples used in each comparison can be seen in S2 Table. The PCA and UMAP were generated with samples that had the complete set of inflammatory markers. (DOCX) [file pntd.0013749.s012.docx]

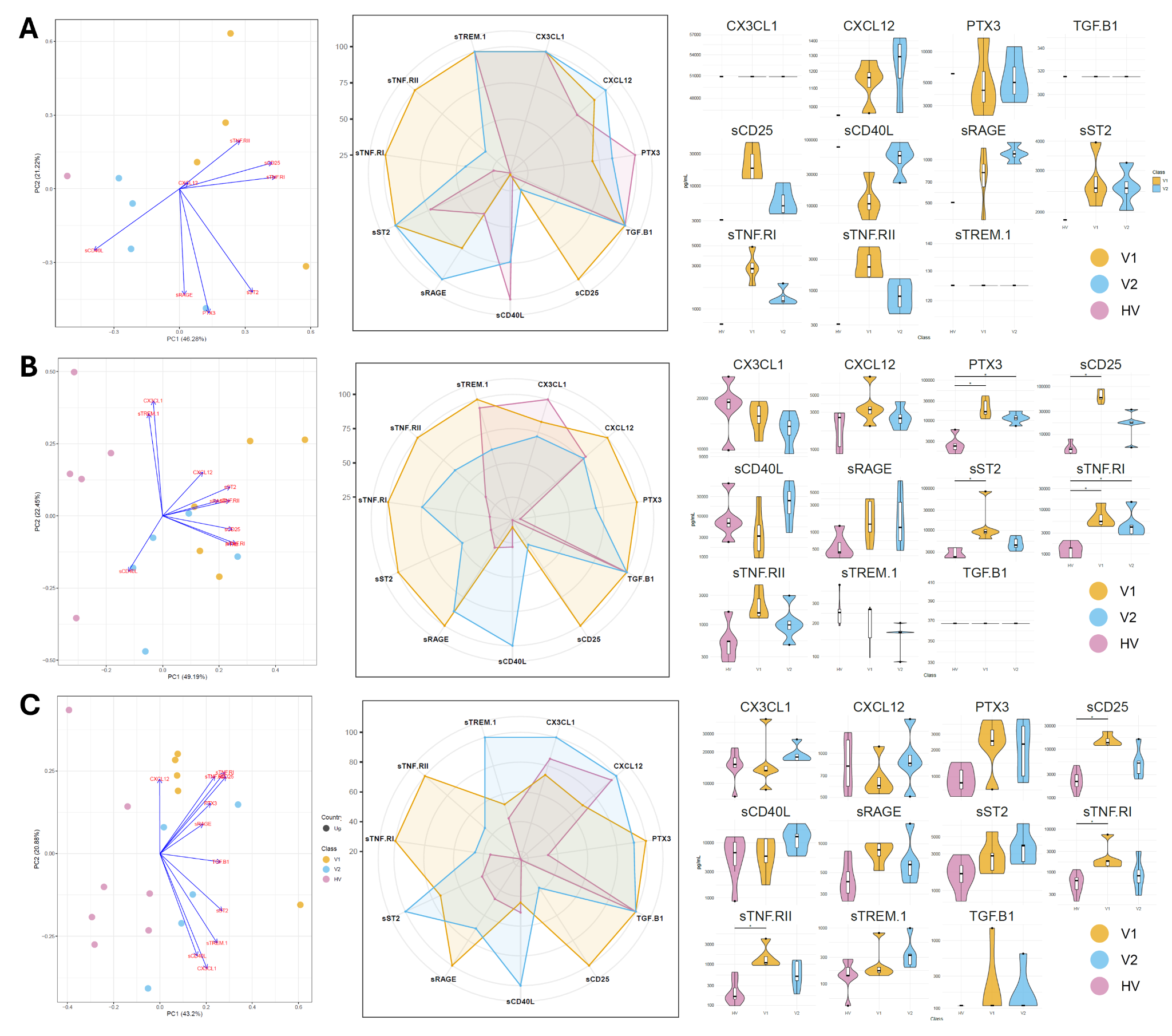


**Supplementary Figure 9: Inflammation immune markers in females across the four countries.** The left panel is a PCA biplot of the inflammation markers values for each patient. Each patient is represented by a dot, coloured in yellow (V1) blue (V2) and red (HV). The arrows represent how much each immune marker contributes to the principal components. The middle panel is a radar plot, where each segment corresponds to the median of patients in a group (V1, V2 or HV), scaled to the highest median in the three groups (ex: if V1, V2 and HV were respectively 20, 10 and 5, they would be represented as 100, 50 and 25) of one inflammation marker. Markers that were significantly (p <0.05) higher in V1 or V2 are respectively coloured in yellow or blue. Markers that were not significantly different are in black. Violin plots showing the range and statistical support for the variations in inflammation markers pre and post treatment. The numbers of asterisks represent p-values, where 1 to 4 corresponds respectively values below 0,05, 0.01, 0.001 and 0.0001. The letters correspond to the results from **A)** Ethiopia, **B)** Kenya, **C)** Sudan and **D)** Uganda. The statistical results and number of samples used in each comparison can be seen in S2 table. The PCA and UMAP were generated with samples that had the complete set of inflammatory markers.
