# Supplementary material for: Systemic inflammatory markers of visceral leishmaniasis treatment response in East Africa
Source: PLoS Negl Trop Dis. 2026 Feb 27;20(2):e0013749. doi: 10.1371/journal.pntd.0013749 (PMC12965683; doi:10.1371/journal.pntd.0013749)
Supplement: S10 Fig — Each panel corresponds to a different trait. Patients are identified by a combination of colour and shape. A) Males Ethiopia, B) Males Kenya, C) Males Sudan, D) Males Uganda. (DOCX) [file pntd.0013749.s013.docx]

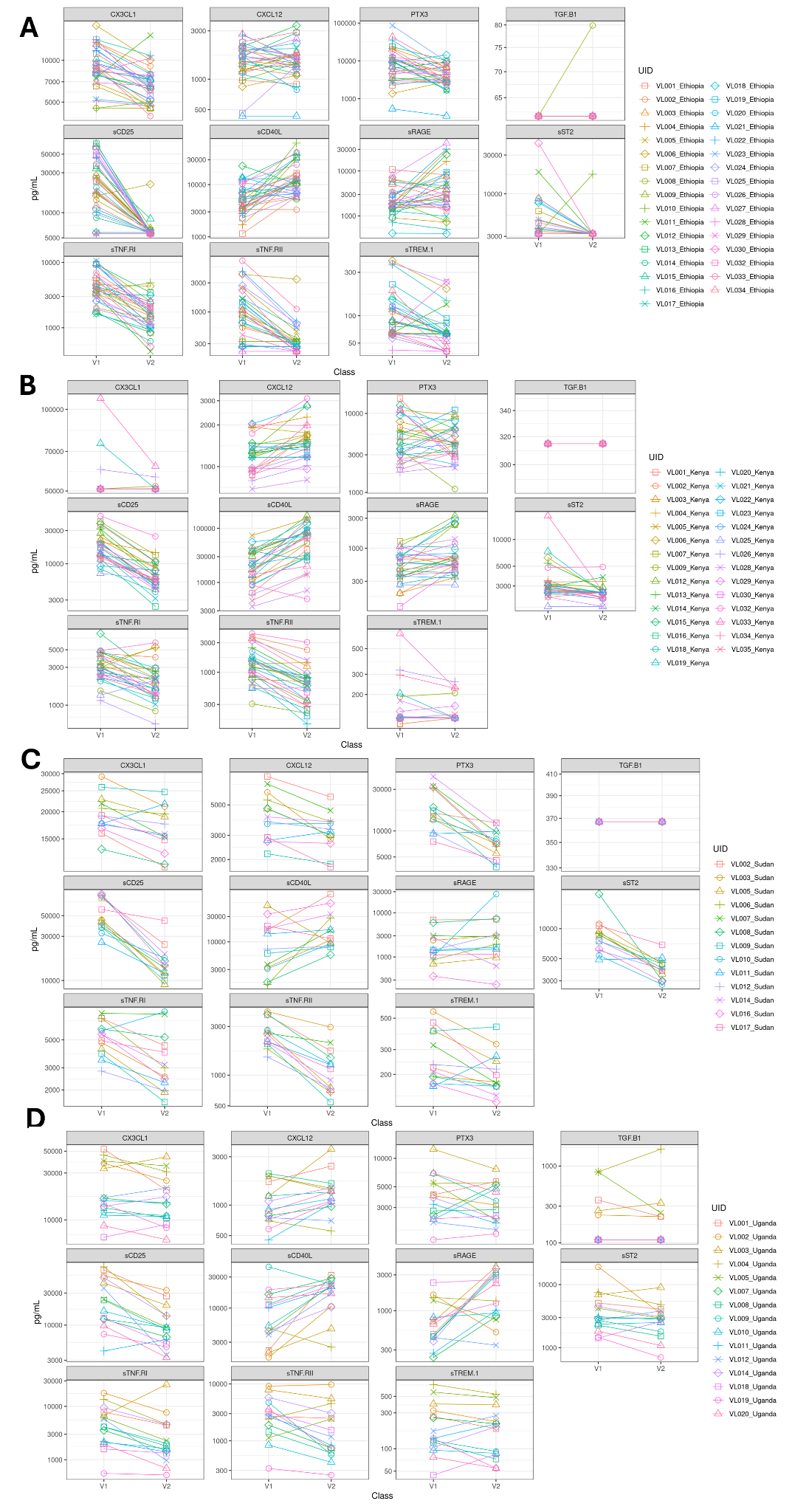


**Supplementary Figure 10: Slopegraph showing male patient’s trait levels before (V1) and after (V2) treatment.** Each panel corresponds to a different trait. Patients are identified by a combination of colour and shape. **A)** Males Ethiopia, **B)** Males Kenya, **C)** Males Sudan, **D)** Males Uganda.
