# Supplementary material for: Systemic inflammatory markers of visceral leishmaniasis treatment response in East Africa
Source: PLoS Negl Trop Dis. 2026 Feb 27;20(2):e0013749. doi: 10.1371/journal.pntd.0013749 (PMC12965683; doi:10.1371/journal.pntd.0013749)
Supplement: S11 Fig — Each dot corresponds to one patient. The X and Y axis represent, respectively, the log-normalized scaled delta (V2 -V2) of sCD40L and Platelet levels. In A) Ethiopia; B) Kenya and C) Sudan. D) Table summarizing the linear regression and Pearson correlation results. Lm_AdjRsquared, Lm_pvalue and Lm_Estimate are respectively the adjusted R squared, p-value and estimate for the logistic regression results, while the Cor_pvalue and Cor_rho represent the p-value and rho from the spearman correlation. Linear regression analysis showed that changes in sCD40L were associated with changes in platelet concentrations for Ethiopia (β = 0.85, R2 = 0.30, p = 0.0006) and Sudan (β = 2,2, R2 = 0.44, p = 0.007), but not Kenya (β = 0.26, R2 = 0.03, p = 0.8). The model explained ~30–44% of the variance in sCD40L concentrations in Ethiopia and Sudan, indicating that while platelets contributed substantially, they were not solely responsible for the variation in sCD40L. (DOCX) [file pntd.0013749.s014.docx]

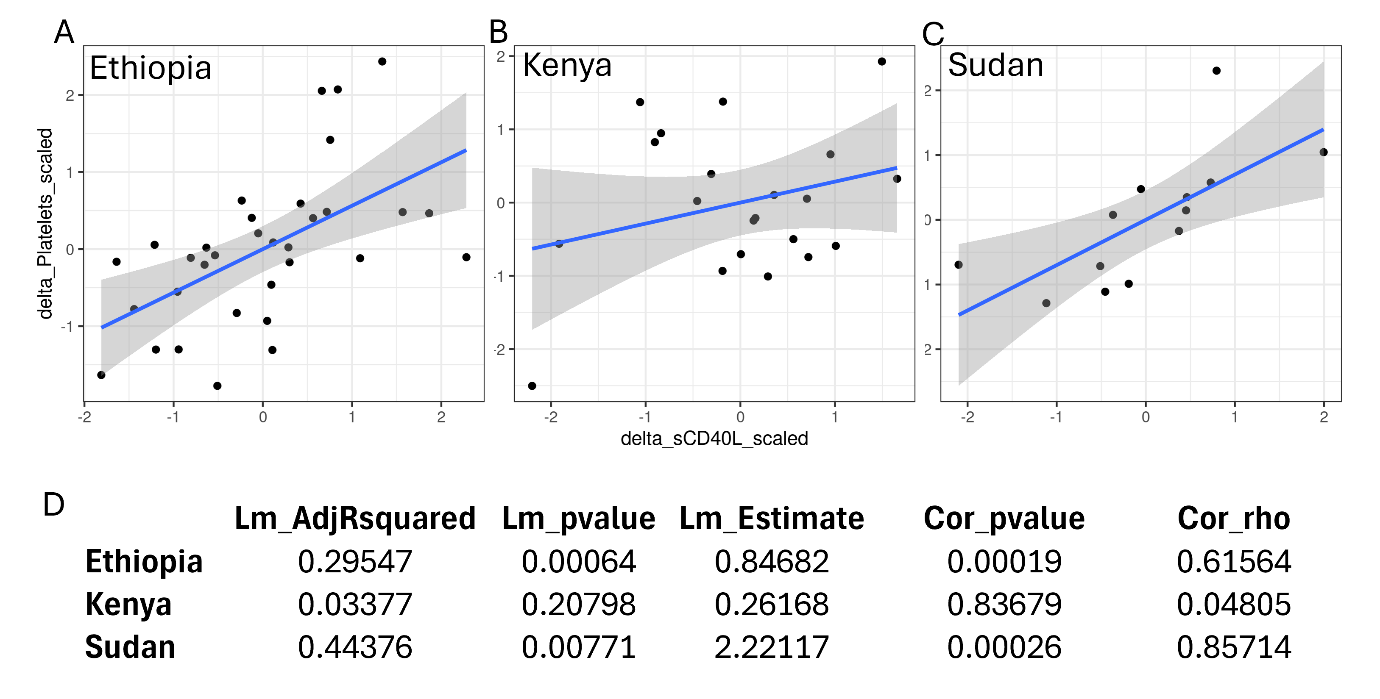


**Supplementary Figure 11:** **Association between variation in sCD40L and Platelet levels before and after treatment**. Each dot corresponds to one patient. The X and Y axis represent, respectively, the log-normalized scaled delta (V2 -V2) of sCD40L and Platelet levels. In A) Ethiopia; B) Kenya and C) Sudan. D) Table summarizing the linear regression and Pearson correlation results. Lm_AdjRsquared, Lm_pvalue and Lm_Estimate are respectively the adjusted R squared, p-value and estimate for the logistic regression results, while the Cor_pvalue and Cor_rho represent the p-value and rho from the spearman correlation. Linear regression analysis showed that changes in sCD40L were associated with changes in platelet concentrations for Ethiopia (β = 0.85, R² = 0.30, p = 0.0006) and Sudan (β = 2,2, R² = 0.44, p = 0.007), but not Kenya (β = 0.26, R² = 0.03, p = 0.8). The model explained ~30-44% of the variance in sCD40L concentrations in Ethiopia and Sudan, indicating that while platelets contributed substantially, they were not solely responsible for the variation in sCD40L.
