# Supplementary material for: Systemic inflammatory markers of visceral leishmaniasis treatment response in East Africa
Source: PLoS Negl Trop Dis. 2026 Feb 27;20(2):e0013749. doi: 10.1371/journal.pntd.0013749 (PMC12965683; doi:10.1371/journal.pntd.0013749)
Supplement: S12 Fig — The left panel shows only correlations with FDR adjusted p-values < 0.05. The right panel shows all of the identified correlations, irrespective of the p-values. A) Ethiopia pre-treatment; B) Ethiopia post-treatment; C) Kenya pre-treatment; D) Kenya post-treatment; E) Sudan pre-treatment; F) Sudan post-treatment; G) Uganda pre-treatment; H) Uganda pos-treatment. (DOCX) [file pntd.0013749.s015.docx]

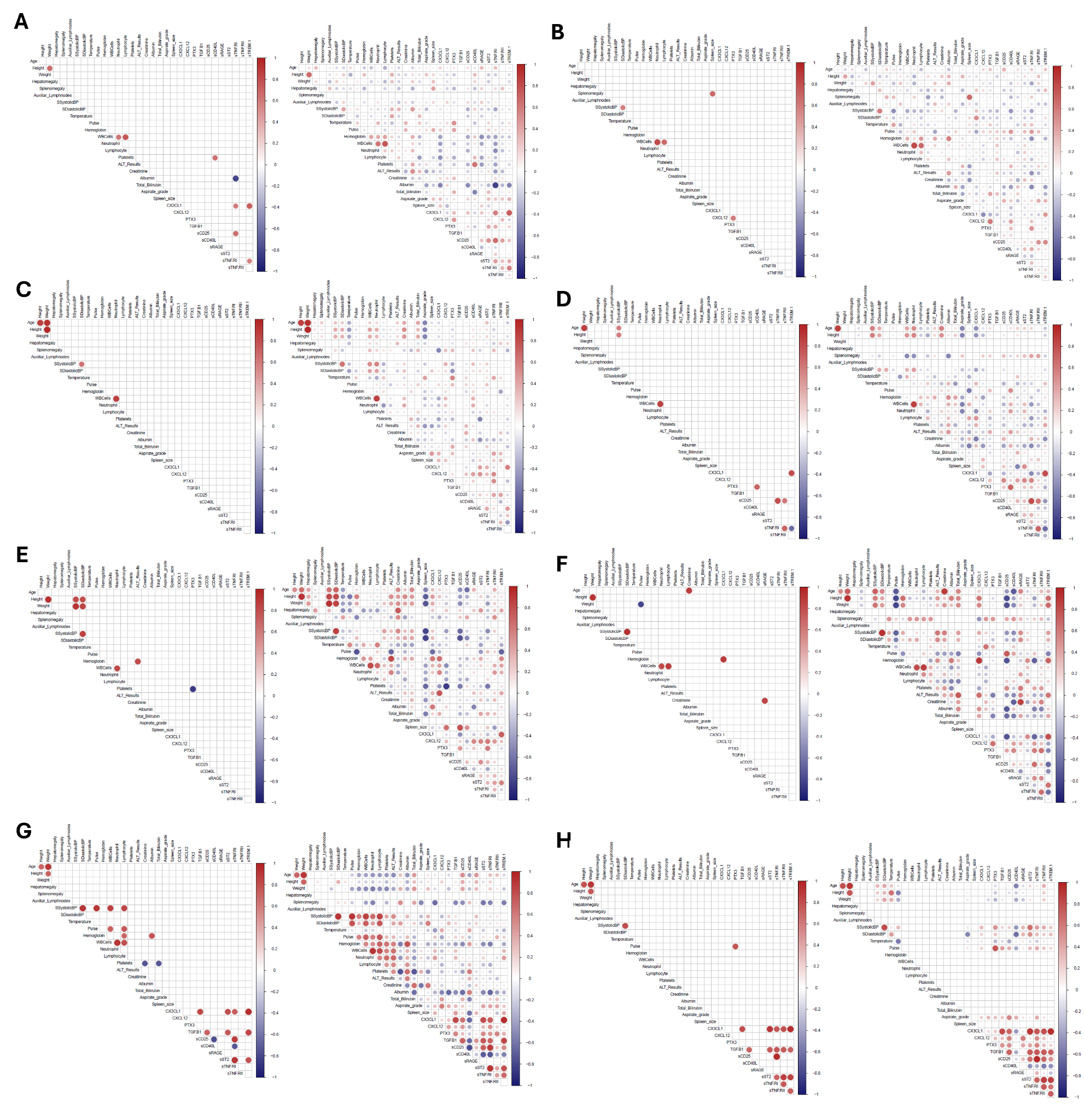


**Supplementary Figure 12: Correlation between clinical, haematological and inflammation markers.** The left panel shows only correlations with FDR adjusted p-values < 0.05. The right panel shows all of the identified correlations, irrespective of the p-values. **A)** Ethiopia pre-treatment; **B)** Ethiopia post-treament; **C)** Kenya pre-treatment; **D)** Kenya post-treatment; **E)** Sudan pre-treatment; **F)** Sudan pos-treatment; **G)** Uganda pre-treatment; **H)** Uganda pos-treatment.
