# Supplementary material for: Systemic inflammatory markers of visceral leishmaniasis treatment response in East Africa
Source: PLoS Negl Trop Dis. 2026 Feb 27;20(2):e0013749. doi: 10.1371/journal.pntd.0013749 (PMC12965683; doi:10.1371/journal.pntd.0013749)
Supplement: S13 Fig — Logistic Regression coefficient with 95% confidence intervals for the association of each marker to the evaluated trait, using Age as a covariate. Increases in values of markers that are above or below zero respectively increases or decreases the Odds Ratio of the trait. Values with p-value <0.05 are presented by triangles. A) Ethiopia hepatomegaly in pre-treatment patients; B) Ethiopia, prediction of persistent splenomegaly based on pre-treatment data; C) Kenya persistent splenomegaly post-treatment. (DOCX) [file pntd.0013749.s016.docx]

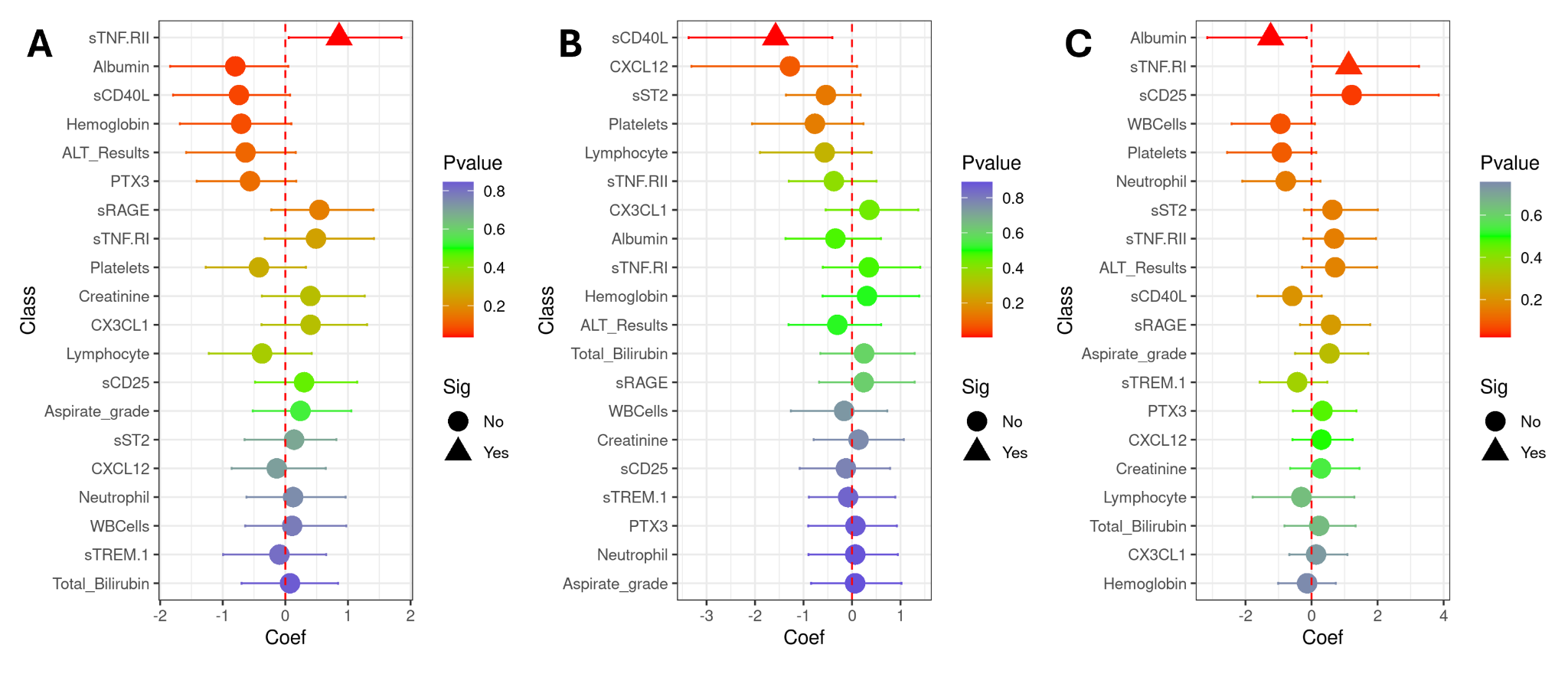


**Supplementary Figure 13: Logistic regression using Age as covariate**. Logistic Regression coefficient with 95% confidence intervals for the association of each marker to the evaluated trait, using Age as a covariate. Increases in values of markers that are above or below zero respectively increases or decreases the Odds Ratio of the trait. Values with p-value <0.05 are presented by triangles. **A)** Ethiopia hepatomegaly in pre-treatment patients; **B)** Ethiopia, prediction of persistent splenomegaly based on pre-treatment data; **C)** Kenya persistent splenomegaly post-treatment.
