# Supplementary material for: Systemic inflammatory markers of visceral leishmaniasis treatment response in East Africa
Source: PLoS Negl Trop Dis. 2026 Feb 27;20(2):e0013749. doi: 10.1371/journal.pntd.0013749 (PMC12965683; doi:10.1371/journal.pntd.0013749)
Supplement: S14 Fig — The left panel corresponds to boxplots showing the logistic regression coefficient values of the 1000 stratified bootstrap replicates. The Y axis represents the markers, and the X axis the logistic regression coefficient. The middle panel corresponds to the result of the logistic regression bootstrap replicates. Each dot corresponds to the result of one of the 1000 replicates, where the X axis represents -log10(p-value) and the Y axis represents the logistic regression coefficient, as a representation of the Odds Ratio. In both left and middle panels, positive and negative values correspond respectively to increased or decreased odds of having the evaluated the clinical outcome. In the middle panel, the vertical and horizontal red lines correspond respectively to the p-value of 0.05 and to the logistic regression coefficient of zero. The right panel corresponds to violin plots comparing hepatomegaly or splenomegaly patients in red (1), with patients without those symptoms in blue (2), using Mann Whitney u test. A) Hepatomegaly in Ethiopia; B) pre-treatment markers prediction of late (post-treatment) splenomegaly in Ethiopia; C) persistent splenomegaly in Kenya. ns: non-significant. (DOCX) [file pntd.0013749.s017.docx]

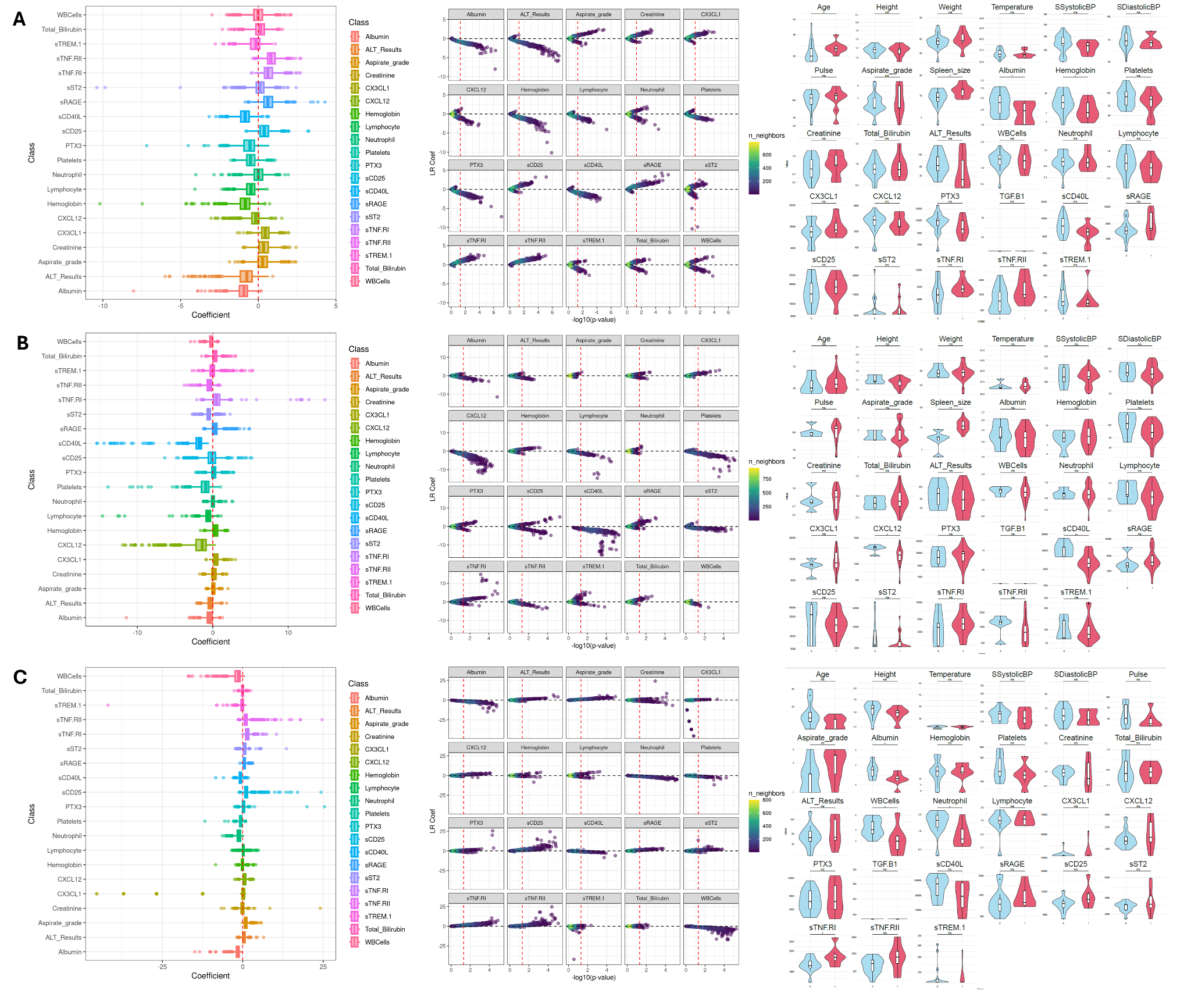


**Supplementary Figure 14: Hepatomegaly and splenomegaly marker assessment.**

The left panel corresponds to boxplots showing the logistic regression coefficient values of the 1000 stratified bootstrap replicates. The Y axis represents the markers, and the X axis the logistic regression coefficient. The middle panel corresponds to the result of the logistic regression bootstrap replicates. Each dot corresponds to the result of one of the 1000 replicates, where the X axis represents -log10(p-value) and the Y axis represents the logistic regression coefficient, as a representation of the Odds Ratio. In both left and middle panels, positive and negative values correspond respectively to increased or decreased odds of having the evaluated the clinical outcome. In the middle panel, the vertical and horizontal red lines correspond respectively to the p-value of 0.05 and to the logistic regression coefficient of zero. The right panel corresponds to violin plots comparing hepatomegaly or splenomegaly patients in red (1), with patients without those symptoms in blue (2), using Mann Whitney u test. **A)** Hepatomegaly in Ethiopia; **B)** pre-treatment markers prediction of late (post-treatment) splenomegaly in Ethiopia; **C)** persistent splenomegaly in Kenya. ns: non-significant.
