# Supplementary material for: Systemic inflammatory markers of visceral leishmaniasis treatment response in East Africa
Source: PLoS Negl Trop Dis. 2026 Feb 27;20(2):e0013749. doi: 10.1371/journal.pntd.0013749 (PMC12965683; doi:10.1371/journal.pntd.0013749)
Supplement: S15 Fig — Top left panel: sPLS-DA loading plot, showing the loading weight of the Inflammation/Clinical markers influence on the assessed trait, ordered from bottom (highest impact) to top (lowest impact). Values in yellow and blue are respectively associated with the presence or absence of the trait. Bottom left panel: Variable Importance in Projection (VIP) of the evaluated Inflammation/Clinical markers for a given trait. Values higher than 1.3 are highlighted in red. Left middle panel: Logistic Regression coefficient with 95% confidence intervals for the association of each marker to the evaluated trait. Increases in values of markers that are above or below zero respectively increases or decreases the Odds Ratio of the trait. Values with p-value <0.05 are presented by triangles. Middle right panel: corresponds to the result of the logistic regression bootstrap replicates. The X axis represents the logistic regression coefficient, as a representation of the Odds Ratio. Positive and negative values correspond respectively to increased or decreased odds of having the evaluated the clinical outcome. Right panel: Model prediction scores results for the PLS-DA leave one patient out (LOPO) cross validation, using increasing numbers of markers, selected based on highest VIP scores. PPV: Positive Predictive Value; NPV: Negative Predictive Value. A) Ethiopia persistent splenomegaly; B) Early hepatomegaly in Sudan; C) predictive power of early markers to identify late splenomegaly in Kenya; D) Early splenomegaly in Sudan; E) Early splenomegaly in Uganda. (DOCX) [file pntd.0013749.s018.docx]

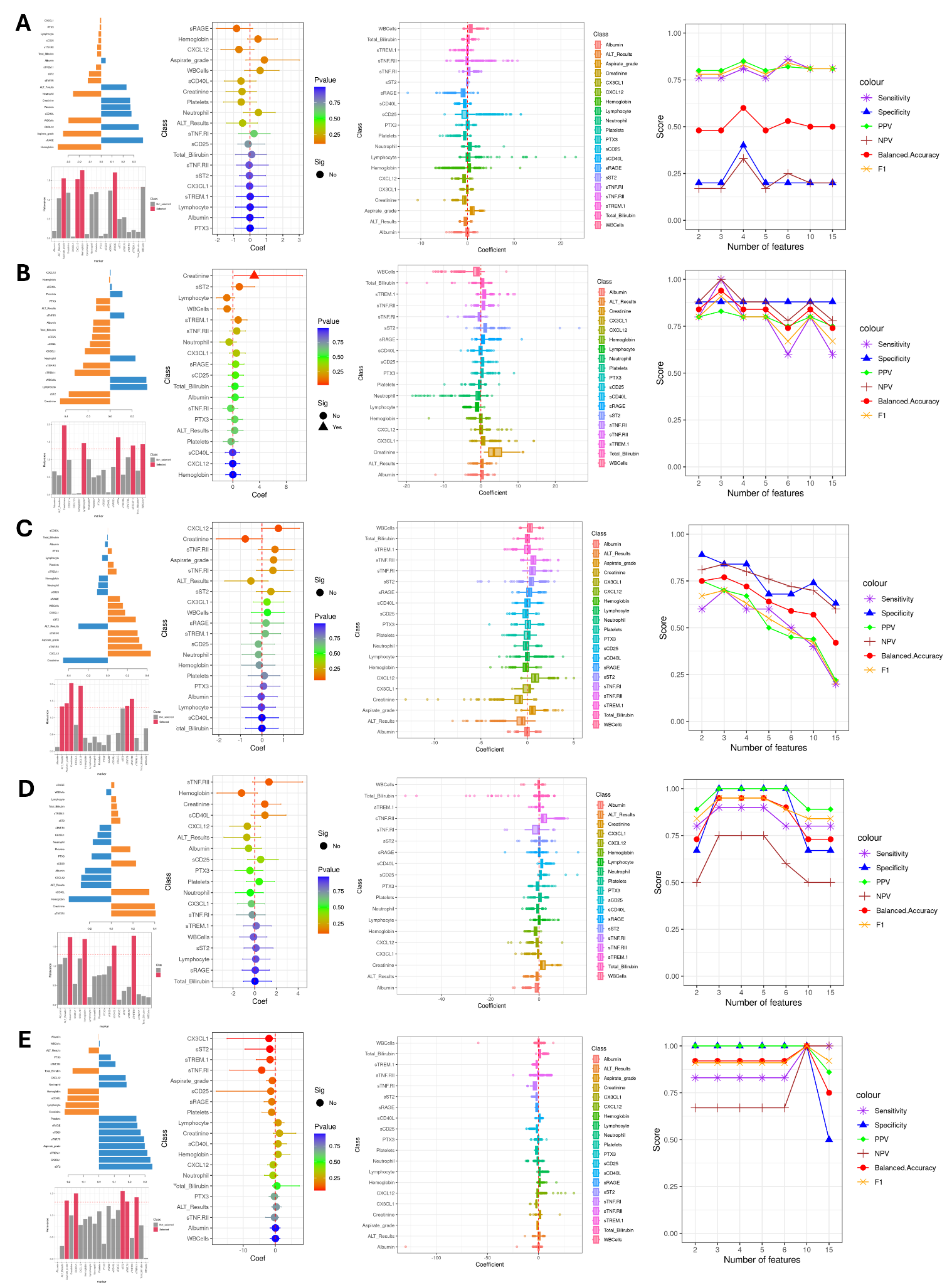


**Supplementary Figure 15: Hepatomegaly and splenomegaly marker assessment. Top left panel**: sPLS-DA loading plot, showing the loading weight of the Inflammation/Clinical markers influence on the assessed trait, ordered from bottom (highest impact) to top (lowest impact). Values in yellow and blue are respectively associated with the presence or absence of the trait. **Bottom left panel**: Variable Importance in Projection (VIP) of the evaluated Inflammation/Clinical markers for a given trait. Values higher than 1.3 are highlighted in red. **Left middle panel**: Logistic Regression coefficient with 95% confidence intervals for the association of each marker to the evaluated trait. Increases in values of markers that are above or below zero respectively increases or decreases the Odds Ratio of the trait. Values with p-value <0.05 are presented by triangles. **Middle** **right panel**: corresponds to the result of the logistic regression bootstrap replicates. The X axis represents the logistic regression coefficient, as a representation of the Odds Ratio. Positive and negative values correspond respectively to increased or decreased odds of having the evaluated the clinical outcome. **Right panel:** Model prediction scores results for the PLS-DA leave one patient out (LOPO) cross validation, using increasing numbers of markers, selected based on highest VIP scores. PPV: Positive Predictive Value; NPV: Negative Predictive Value. **A)** Ethiopia persistent splenomegaly; **B)** Early hepatomegaly in Sudan; **C)** predictive power of early markers to identify late splenomegaly in Kenya; **D)** Early splenomegaly in Sudan; **E)** Early splenomegaly in Uganda.
