# Supplementary material for: Systemic inflammatory markers of visceral leishmaniasis treatment response in East Africa
Source: PLoS Negl Trop Dis. 2026 Feb 27;20(2):e0013749. doi: 10.1371/journal.pntd.0013749 (PMC12965683; doi:10.1371/journal.pntd.0013749)
Supplement: Attachment — Submitted filename: Leishmaniasis REVIEW.docx [file pntd.0013749.s025.docx]

REVIEW of PNTD-D-25-02021 “Systemic inflammatory markers of visceral leishmaniasis treatment response in East” by Ayenew Addisu et al.

East Africa is now the primary hotspot of visceral leishmaniasis (VL) globally. As the authors have stated “There is substantial variation among symptomatic cases regarding disease progression, response to treatment, relapse and occurrence of post-kala-azar dermal leishmaniasis (PKDL).” The authors have sought to identify biological markers of response to treatment, in particular of hepatosplenomegaly. The researchers have conducted an observational study in four East African countries; 40 VL patients and 30 “convenience” controls from each of four countries. However, *not all countries were able to meet the planned sample sizes*; the final sample sizes were 109 VL patients (as opposed to 160) and 103 controls (as opposed to 120)*.*

The standard first-line of treatment using the combination of sodium stibogluconate (SSG) and paromomycin (PM) as recommended by the WHO was adopted. For a few patients, a departure from this line of treatment had to be followed because of their slightly different course of disease progression compared to the others. The various sites participating in this study adopted same strategies of testing for infection and follow-up phenotyping, including tests (FACS) for 11 markers of inflammation. *Adoption of this uniform strategy is a strength of this study.*

The impact of treatment on hepatomegaly was significant; most patients recovered. However, the impact on splenomegaly was highly variable. Recovery of hematological parameters was also high, although the proportions of patients with parameters in the normal reference range at the time of recruitment were variable across countries (possibly indicating that levels of infection at recruitment were non-uniform). Ethiopia had the worst profile of parameters compared to patients from the remaining countries; a large proportion of patients sample from Ethiopia was outside of the normal reference range. However, the patients from Ethiopia showed the best outcome of treatment.

*This study has identified markers that significantly correlate with improvement after treatment of the various parameters of infection progress and phenotypes resulting from infection. However, because of the restricted sample size, the identities of the markers can at best be viewed as tentative. The data generation and analyses have been competently carried out and some important conclusions have been drawn.*

**I recommend that the manuscript be published.** However, I have found the manuscript to be too long with many graphs and descriptions that are tangential to the main theme of this research. It is difficult for me to pinpoint these, but **I request the authors to substantially reduce the length of the manuscript to at most two-third of its present length, but preferably to half.**
