# Supplementary material for: Systemic inflammatory markers of visceral leishmaniasis treatment response in East Africa
Source: PLoS Negl Trop Dis. 2026 Feb 27;20(2):e0013749. doi: 10.1371/journal.pntd.0013749 (PMC12965683; doi:10.1371/journal.pntd.0013749)
Supplement: Attachment — Submitted filename: Addisu et al_PLOSNTD_Response_to_reviewers.docx [file pntd.0013749.s026.docx]

**Overall comment for all reviewers/Editor:**

We would like to thank the reviewers for their positive comments and valuable considerations to improve our manuscript. As suggested, we have shortened the manuscript ~10-15%, including fully removing figure 5; and increased the font size and resolution of the main figures to increase clarity. We have also included text / references in the Introduction further describing VL immune characterization in EA, regarding human heritage variability in East Africa and about the selection of the target markers. We have also reinforced that our VL marker assessment was exploratory, rather than definitive. We hope that with these changes, the reviewers and editor agree that the manuscript is suitable for publication in *PLOS Neglected Tropical Diseases*. The response for each point raised by the reviewers is below. In this response, the line numbers refer to the manuscript version with tracked changes.

**Reviewer 1:**

(…) I recommend that the manuscript be published. However, I have found the manuscript to be too long with many graphs and descriptions that are tangential to the main theme of this research. It is difficult for me to pinpoint these, but I request the authors to substantially reduce the length of the manuscript to at most two-third of its present length, but preferably to half.

**Response:** We thank the reviewer for the positive comment. As requested, we have shortened the manuscript, removing, among others, redundant parts of some figures and the Figure 5 in its entirety. However, as requested by reviewer 2, we had to include some new information in the Introduction and Discussion. This resulted in an overall 10-15% reduction in the manuscript size. We hope that this more concise version satisfies the reviewer’s request.

**Reviewer 2:**

(…) Overall, the study provides valuable descriptive data from four East African regions where VL burden is high. It documents consistent improvements in clinical and inflammatory markers at the end of treatment. However, the predictive components should be toned down and contextualized as exploratory rather than definitive.

1. A significant limitation of the study, as acknowledged by the authors, is the relatively low number of samples per country. The sample size becomes even smaller when cases with incomplete data are excluded from downstream analyses. This limitation is further compounded by substantial heterogeneity in clinical and immunological profiles across the four regions, which necessitated analyzing each country separately. The authors should discuss this limitation more explicitly and support it with appropriate references to similar epidemiological or biomarker studies that faced comparable sample size constraints. Another important limitation is the skewed sex distribution in the cohort, which is predominantly male. The authors should address whether and how the findings can be generalized to females, and discuss any known sex related differences in VL presentation or immune responses

2. The authors also attempt modelling approaches such as logistic regression and PLS DA to identify blood based markers predicting enlarged spleen or liver at baseline or incomplete recovery after treatment. However, because these analyses use only patients with complete data, the sample sizes become even smaller in some countries. The authors acknowledge the lack of robustness in predictive modelling under these constraints. Hence, these claims should be interpreted with caution.

**Response to 1 and 2**:

**Response:** We thank the reviewer for these important considerations. We agree with the reviewer that our sample size is limiting for complex modelling as PLSDA and, to some extent, logistic regression. We tried to be very clear regarding the “exploratory” characteristics of the marker identification (PLSDA – Logistic regression) in the initial submitted version of the manuscript (lines 450-452; “*Given the small and unbalanced sample sizes, these markers should be interpreted with caution, rather than definitive predictors of these outcomes*”) and lines 582-584; “*Model building was challenging due to* *the limited sample size, imbalanced case/control distribution, and significant heterogeneity across patients*”). To reinforce this further, we have added an additional line to the Discussion (new text, lines 584-585; “*Hence, the identified markers are only exploratory, and would benefit from future validation*”).

However, we do believe that for the simpler paired Wilcoxon, our sample size is adequate and in the range of other publications in the literature that evaluated immune markers in in visceral leishmaniasis, such as:

1-Santos 2016 (doi:10.1371/journal.pntd.0004375), published in *PLOS NTD*: Evaluated the variations in cytokines in patients from Sergipe Brazil, using the Human Th17 Kit, Merck Millipore. Sample size: 65 patients, which were divided into four groups with sizes from 11 to 25 patients.

2-Tadesse 2021 (DOI: 10.1371/journal.pntd.0009632), published in *PLOS NTD*: Evaluated IL-10, IFN-γ, TGF-β1, and IL-2 concentrations in sera/plasma of VL patients in Ethiopia. Sample size: 48 patients, with different treatment regimens: 35 with SSG/PM, 11 with only SSG.

3-Dijk 2024 (DOI: 10.1017/S0031182024000751): published in *Parasitology*: Evaluated blood cytokine levels in VL patients in Kenya, before and after SSG-PM treatment. Sample size: 20 confirmed VL patients.

4-Hailu 2005 (DOI: 10.1016/j.clim.2005.06.015): Published in *Clinical Immunology*: Evaluated the IFN-g, IL-4, or IL-10 levels and CD8 and CD8 profile in VL patients with different clinical stages of the disease. Sample size: 33 VL patients, 9 treated VL patients, 14 subclinical infections, 34 asymptomatic infections and 18 healthy controls.

We have included further description of some of these studies in the Introduction (new text, lines 143-151; “*Most clinical studies evaluating patients at admission and end of treatment (EoT) in EA have been restricted to one country and used limited marker panels. In Ethiopia, IL-10 and IFNγ were identified as potential markers for active VL, with IL-10 concentration declining within seven days of treatment [38]. In Kenya, severe VL was also associated with higher circulating IL-10 and IFNγ, with reductions in both at 17 days post treatment with SSG/PM, despite persistent anaemia and circulating IL-17 [39]. In Sudan, a longitudinal study identified IFNγ and TNF concentrations as important markers for active VL. [40]. Hence, the broader systemic immune / inflammatory status of patients across EA countries at the end of treatment with SSG/PM is not fully understood.”)*.

Regarding the male/female unbalanced dataset, this is a common observation in VL studies, and was briefly pointed out in the Introduction (lines 117-119; “*There is also evidence of gender disparity in VL cases, which might relate to behaviour and/or biological differences between sexes* [*[21–23]*](https://paperpile.com/c/KTTF1f/XAIV+zs55+M9k4)”) We did analyse all data from females and described comparisons with data from males when possible (lines 371-373; “*The limited female sample size also reduced the statistical power for male–female comparisons, where only higher creatinine concentrations in males pre-treatment (V1) from Sudan reached statistical significance (S6 Figure)*”, lines 396-397; “*Comparable tendencies were observed for female patients (S9 Figure), although with lower statistical support.*”; and lines 407-410: “Comparisons between males and females were again limited by the small female sample size. However, PTX3 concentration was significantly higher in males after treatment in Sudan, whereas sRAGE concentration was higher in females following treatment in Uganda (S6 Figure)”). Female data were also provided in Supplementary Figure 6.

3. In subsection 2.6 the authors state that analyses were performed in R but do not provide package versions, seeds, or code availability. Methods such as UMAP require reporting of parameters including seed, n neighbors, min dist, and metric because of their stochastic nature. Even PCA and variable selection criteria should be more clearly described. The authors should specify which clinical markers were included in PCA and UMAP, justify the choices, and clarify how missing Uganda V2 data were handled.

**Response to 3**: We thank the reviewer for the meticulous evaluation of the manuscript. As requested, we have included a detailed description of the UMAP parameters in the methods section 2.6 (new text, lines 310-311: “*The UMAPs were generated using the following parameters: min_dist = 0.1, metric = "euclidean", n_epochs = 1000, n_neighbour = 15, seed=123*”). In the previous UMAP plots, we did not use a fixed seed, and although there were changes in the layout, the V1 – V2 – HV group separation was always supported. We have now fixed the seed as “123” and updated the UMAP plots. Regarding the PCA, even though not explicitly show in the main text most of its characterization (including the scree plot with the relevance of each PC) was provided in Supplementary Figure S3.

We would like to reiterate that the code availability was described in the initial submission of the manuscript under Data and code availability (lines 666-670; “*The R script used for data analysis can be obtained from GitHub: https://github.com/jaumlrc/Immstat_cure.git”). We have now also included a list with all the packages and versions in the GitHub page*.

Regarding the UMAP description, only immune markers and not clinical markers were used in the UMAPs in Figure 3, as described in the Methods (lines 312-314: “*For the country specific Inflammation data PCA/UMAPs, the used markers were: CX3CL1, CXCL12, PTX3, TGF.B1, sCD25, sCD40L, sRAGE, sST2, sTNF.RI, sTNF.RII and sTREM.1”*. Clinical markers were only used in the PCA in Figure 5, which has now been removed from the manuscript.

Regarding the missing Uganda V2 samples, these samples were only missing the clinical/haematological results, but they had measurements for all immunological markers. Hence, they were included in the UMAPs in Figure 3.

4. The interpretation that increased sCD40L at end of treatment indicates restored immune homeostasis is not fully supported. Because sCD40L levels closely track platelet activation and platelet abundance, the rise in sCD40L may simply reflect platelet recovery rather than improved anti leishmania immunity. Since platelet counts also increase in parallel especially in the Sudan cohort, normalizing sCD40L to platelet number would prevent over interpretation of platelet driven changes.

**Response to 4**: This is an excellent comment, which led us to estimate the association between variations in sCD40L and Platelet levels (see below). However, we would like to reiterate that the sentence in the result section regarding “restored homeostasis” was considering not only in relation to sCD40L, but primarily the large reductions in sTNFRI and II after treatment. Several of the severe symptoms of VL, including splenomegaly and pancytopenia, are influenced by levels of immune mediators as TNF. It is also important to note that as the pancytopenia (and consequently the thrombocytopenia) is at least partially immune mediated in VL, and it is reasonable to speculate that the increase in platelet levels could be an indirect outcome of partial return to immune homeostasis.

As pointed out by the reviewer, platelets are one of the main sources of sCD40L. To investigate this further, we evaluated the association between sCD40L and platelet changes in V1 to V2, using logistic regression and spearman correlation. To that end, for each patient, for the sCD40L and Platelet traits, we log-standardized the result and obtained the V2-V1 delta, which was scaled and centred. Then, we estimated the linear regression between the delta_sCD40L and delta_Platelets levels. This analysis showed that, as expected, both are largely correlated, where the variation in platelet levels partially explains the variations in sCD40L, as in the figure below:

***Association between variation in sCD40L and Platelet levels before and after treatment****. Each dot corresponds to one patient. The X and Y axis represent, respectively, the log-normalized scaled delta (V2 -V2) of sCD40L and Platelet levels. In A) Ethiopia; B) Kenya and C) Sudan. D) Table summarizing the linear regression and Pearson correlation results. Lm_AdjRsquared, Lm_pvalue and Lm_Estimate are respectively the adjusted R squared, p-value and estimate for the logistic regression results, while the Cor_pvalue and Cor_rho represent the p-value and rho from the spearman correlation.*

sCD40L and platelet deltas were highly correlated in Ethiopia (~0.6) and Sudan (0.9), but not in Kenya (0.04). Also, the linear regression model explained ~30 and 44% of the variance in sCD40L for Ethiopia and Sudan, but only ~3% in Kenya. This indicates that platelet levels contribute substantially but not fully explain sCD40L variation. As there are no platelet levels for V2 in Uganda samples, it was not evaluated in this analysis.

We have included the data above as new Supplementary Figure 11 and refer to this figure in line 403-405. It is worth noting, as pointed out in the discussion, that high sCD40L levels were associated with better prognostic for VL patients, with and without HIV coinfection in other studies. Recombinant sCD40L was also shown to reduce macrophage infection and intracellular amastigote load, showing that it might be relevant in VL control, so it is relevant for VL, regardless of the origin.

Minor Comments
1. Some modelling terms such as ‘dim1’ and ‘dim2’ can be unfamiliar to readers without a statistical background. Including brief definitions within the text or figure legends would help. Similarly, abbreviations such as SSG PM and HV should be defined at first use. For figures that include multiple immunological markers (for example, sTNFRI, sRAGE), the authors may include full marker names or short biological descriptors either in the figure legend or in a supplementary table for easier interpretation by a general audience.

**Response**: We thank the reviewer for their comment. We have change the ‘dim1’ ‘dim2’ to UMAP1 and UMAP2, to be in accordance with the common nomenclature. We have also defined all abbreviations on their first appearance. Regarding the immunological markers, we included the full names in section 3.3 (new text, lines 380-385). Regarding their biological relevance, we have noted the biological relevance of the relevant immune markers in the Discussion and included additional references in the Introduction (lines 165-167) .

2. The rational for choosing the inflammatory markers should be properly discussed in the introduction section citing appropriate references.
**Response**: To better characterise the inflammatory marker selection, we made several changes to the Introduction.

1- We extended the text regarding other studies that measured cytokine markers in EA, to better characterize the “state of the art” for VL patient immune markers in EA (new text, lines 143-151; “ *Most clinical studies evaluating patients at admission and end of treatment (EoT) in EA have been restricted to one country and used limited marker panels. In Ethiopia, IL-10 and IFNγ were identified as potential markers for active VL, with IL-10 concentration declining within seven days of treatment [38]. In Kenya, severe VL was also associated with higher circulating IL-10 and IFNγ, with reductions in both at 17 days post treatment with SSG/PM, despite persistent anaemia and circulating IL-17 [39]. In Sudan, a longitudinal study identified IFNγ and TNF concentrations as important markers for active VL. [40]. Hence, the broader systemic immune / inflammatory status of patients across EA countries at the end of treatment with SSG/PM is not fully understood*”)

2-We further described Immstat@cure, to reinforce that this is only the first publication in a series that will characterize in detail several aspects of the VL immune response in EA at admission and EoT (new text, lines 156-162; “*The ImmStat@cure consortium encompassed four EA countries (Ethiopia, Kenya, Uganda and Sudan) representing regions with the highest VL prevalence, and genetically diverse host populations [41,42]. It aimed to characterize patient immune response by several approaches, including serologic immune markers, T and B cell immunophenotyping, transcriptomics and proteomics.*”).

3-To justify our panel, we have included some references that characterize the biological relevance of the markers in LEGENDplex Inflamation panel 2 (lines 165-167). Most of the previous analysis in EA focused on IFN-y and IL10. The markers used here are complementary and expand on what is already known for VL in EA. We hope that these changes are adequate to justify the panel evaluation.

3. The treatment protocol (dose/duration etc.) should be clearly mentioned in the methods section.

**Response**: We thank the reviewer for spotting this omission. We have included the following statement on the Methods (new text, lines 221-222; “Patients were treated with SSG (20mg/Kg/day; i.m.) and PM (15 mg/kg/day ; i.m.) for 17 days”). This is current standard of care for VL patients in EA.

4. The authors should also clarify whether a random sampling strategy was adopted, and if multiple participants were enrolled from the same household or family, they should discuss how potential hereditary biasness was avoided.

**Response**: The recruiting sampling strategy as well as inclusion and exclusion criteria are detailed in the Protocol (S1_Supplementary_Information_file.pdf). Patients were recruited by active case detection and self-attendance at study hospitals. Samples were anonymized, and it was not evaluated if they came from the same household. We have included the additional statement (new text, line 193-194; “*Patients were recruited by active case detection and self-attendance at study hospitals*”).

5. Axis labeling in most of the figures are too small and are difficult to read. The font size should be increased for better readability.

**Response**: We have increased the font size for most figures as requested.

**Reviewer 3:**

1. The concern I have is that the rationale of studying those four populations is missing. Ethiopia, Kenya, Uganda, and Sudan are genetically diverse regions reflecting ancient migrations and linguistic groups (Afro-Asiatic, Nilo-Saharan, Bantu), with East Africa showing deep roots, high mtDNA and other autosomal haplogroup variability. I would suggest to include this point in the introduction section. The differences in outcome may stem from the differences in the genetic make-up of the unique populations studied.

**Response**: We agree with the reviewer that the genetic background of the host influences the VL outcome. Although we had not specifically addressed the EA genetic diversity in the Introduction, we had acknowledged its potential impact (lines 112-113; “*Host and parasite genetics are likely important factors for disease severity [12–14], including polymorphisms in the HLA locus [13,15,16]*”).

We have also included additional information on the study design (new text, lines 156-162**;** “*The ImmStat@cure consortium encompassed four EA countries – Ethiopia, Kenya, Uganda and Sudan – representing regions with the highest VL prevalence, and genetically diverse host populations*”), citing references [[41,42]](https://paperpile.com/c/otjP6M/XjCs+RBWG) that characterize ancient migrations and settlers that generated the recent populations in EA. We did not collect human genetic information for this project due to ethical limitations. Hence, any inference regarding ancestry and host genetics would be speculative.

2. Authors mentioned that Leishmaniasis patients exhibit co-infection with HIV. It would be useful to have some comparative information on inflammatory markers in VL and VL-HIV patients.

**Response**: We thank the reviewer for this consideration. The study of HIV coinfected patients was outside of the scope of the current project, and as the manuscript is already long, we would prefer not to include discussion on this point. We briefly described HIV patients and relapse markers in the Introduction (lines 113-117; *“In EA, the highest incidence of symptomatic VL is observed among malnourished children and young adults, and HIV co-infected patients, as a consequence of both immunological naivety/suppression and behaviours that increase exposure to Phlebotomus vectors in endemic settings [17–20]*”, and regarding relapses (lines 121-126) “*VL relapses are often associated with HIV co-infection. In Ethiopia, more than 50% of the VL-HIV coinfected patients relapse within 9 months of the end of the treatment [24], maintaining high parasite loads, hepatosplenomegaly and pancytopenia at the end of treatment cycle [25]. VL-HIV relapses were associated with failure to restore antigen-specific production of IFN-γ, persistent lower CD4+ T cell counts and higher expression of PD1 on CD4+ and CD8+ T cells [25].*”).

3. The discussion is too lengthy. It needs to be succinct.

**Response**: We thank the reviewer for this consideration. We agree that the manuscript was long. Hence, we removed some redundancy from the Results section, including Figure 5 and reduced some of the overly descriptive text in the Discussion. This resulted in a reduction in word counts of ~10% for the whole manuscript, and 15% in the Discussion, even after the inclusion of the requested modifications by Reviewer 2.
